# Parallel Pathways, Divergent Outcomes: Adipose Tissue–Neural Crosstalk in Depression and Obesity

**DOI:** 10.3390/jcm14238307

**Published:** 2025-11-22

**Authors:** Andrei Prodaniuc, Cornelia Amalinei, Irina Draga Caruntu, Adriana Laura Riscanu, Adriana Grigoraș

**Affiliations:** 1 Department of Morphofunctional Sciences I, Grigore T. Popa University of Medicine and Pharmacy Iasi, 700115 Iasi, Romania; andreiprodaniuc@yahoo.com (A.P.); irina.caruntu@umfiasi.ro (I.D.C.); laura_knieling@yahoo.com (A.L.R.); 2 Department of Histopathology, Institute of Legal Medicine, 700455 Iasi, Romania; 3 Romanian Medical Science Academy, 030171 București, Romania

**Keywords:** depression, adipokines, adipose tissue, obesity, hypothalamic–pituitary–adrenal axis, brain-derived neurotrophic factor, gut–brain axis, cytokines, serotonin, adipokines-based therapy

## Abstract

Depression and obesity are amongst the most serious global health challenges. Each of them is associated with high morbidity, chronicity, and socioeconomic burden. Increasing evidence suggests that these conditions are not merely comorbid but share convergent biological pathways (e.g., hypothalamic–pituitary–adrenal axis dysregulation, chronic inflammation, gut dysbiosis, and mitochondrial dysfunction). All these components contribute together to the development and persistence of depressive symptoms as well as to an increase in adiposity. Within this framework, adipose tissue has emerged as an essential endocrine organ that has a deep impact on neuroimmune signalling and mood regulation through its secreted molecules, such as leptin, adiponectin, resistin, omentin, apelin, chemerin, and visfatin. The current management of depression involves a comprehensive, multidisciplinary approach that includes pharmacological treatment and psychotherapeutic support, alongside lifestyle changes. Here we highlight the molecular crosstalk between adipose tissue and the brain, summarising the evidence of adipokines’ dysregulation role in connecting metabolic dysfunction to depressive neurobiology. By integrating metabolic, immunological, and neuroendocrine perspectives, this narrative review underscores the need to reconceptualise depression as an immunometabolic disorder. Understanding adipokine-mediated pathways may reveal new biomarkers and therapeutic targets, fostering interdisciplinary approaches. This would allow for the development of new treatment strategies, which include recombinant adipokines, anti-inflammatory agents, and microbial modulation. These new strategies might provide a significant benefit in selected patients, in addition to conventional antidepressants.

## 1. Introduction

Depressive disorder remains one of the most prevalent and disabling psychiatric conditions around the world [1]. Its burden extends beyond health, as depression causes serious social and economic consequences as well [1]. As a chronic disorder, depression follows a recurrent course, in which patients experience at least one more episode during their lifetime [1,2]. Such clinical characteristics underscore the urgent need for a deeper understanding of depression’s neurobiology. However, despite decades of research, depression’s pathophysiology is only partially understood. The traditional monoaminergic hypothesis fails to explain the complexity of its aetiology, clinical manifestations, and course [3].

Depression was initially thought to only be caused by psychosocial events. As research progressed, our understanding of depression’s pathogenesis extended beyond the psychosocial theory, allowing clinicians and researchers to comprehend it from a neurobiological perspective. Chronic stress, a main psychosocial contributor to depression, acts as a strong activator of the hypothalamic–pituitary–adrenal (HPA) axis, which, in turn, leads to hypercortisolaemia that hinders therapeutic outcomes [4]. Moreover, the exaggerated HPA axis response contributes to a state of chronic low-grade inflammation, characterised by increased pro-inflammatory cytokines (e.g., IL-6, TNF-α, and IL-1β) [3,5]. Additionally, emerging evidence support the existence of a brain–adipose–gut axis [6,7,8]. Dysbiosis and increased intestinal permeability in obesity contribute to further immune activation, while microbial metabolites modulate neurotransmission and neuroinflammation [7,9]. Similarly, mitochondrial dysfunction disrupts neuronal energy metabolism and oxidative balance [10,11,12]. All things considered, depressive disorder exceeds its clinical definition as a mere psychiatric illness, representing a multisystem disorder that involves endocrine, neuroimmune, and metabolic axes.

Recently, adipose tissue has been proposed as a key component in the interplay between depression and obesity [10,13]. Formerly regarded as a passive fat reservoir, adipose tissue is now recognised as an active and dynamic endocrine organ. It secretes numerous adipose-derived factors, collectively known as adipokines. These bioactive molecules (e.g., leptin, adiponectin, apelin, resistin, omentin, visfatin, and chemerin) are potent metabolic regulators that can regulate appetite, glucose metabolism, immune response, inflammatory signalling, and cardiovascular function [10,13]. Additionally, adipokines can cross the blood–brain barrier (BBB) and interact directly with neuronal receptors. Therefore, adipokines are able to influence neuroplasticity, neurogenesis, and neurotransmission, in addition to their clear metabolic effects [10,13]. For this reason, they can be regarded as true neurometabolic mediators that connect peripheral energy status to central mood regulation [11].

Analogous to depression, obesity has emerged as another serious global health problem [13]. Its complexity lies in obesity’s multifactorial nature that includes genetic, metabolic, behavioural, and environmental factors [13]. There is a significant bidirectional relationship between depression and obesity, which is defined as a body mass index (BMI) ≥ 30. In this respect, increased adiposity can raise depressive risk by nearly two-thirds, whereas depression heightens weight-gain predisposition [14,15]. This link emerges early in life, as childhood obesity increases the likelihood of youth depression, given that both diseases share lifestyle and psychosocial risk factors [16,17]. Such observations suggest that each disorder may result from the other and may aggravate each other, highlighting the existence of common biological mechanisms between mood and energy homeostasis. In this context, the obesity–depression link is supported by mounting evidence, since both conditions are characterised by systemic inflammation and dysregulated neuroendocrine stress systems [4,18,19]. The hypothalamic–pituitary–adrenal (HPA) axis is commonly hyperactive in depression, yet the consequent hypercortisolaemia is not only responsible for altered brain function, but also for metabolic disturbances, such as insulin resistance and increased adiposity [4,18,19]. Conversely, obesity is associated with increased IL-6, TNF-α, and IL-1β levels, creating a pro-inflammatory state that could contribute to depressive symptoms [10,20,21]. Additionally, dysregulation of adipokine secretion, commonly found in obesity, may thus link metabolism to psychic processes, representing a main contributor to the crosstalk between the brain and adipose tissue [11]. Gut dysbiosis also promotes energy extraction that ultimately favours adiposity [22]. Thus, obesity’s effects transcend somatic comorbidities and extend into the field of mental health, shaping a bidirectional association with depression, which cannot be explained only by psychosocial theories [23,24]. In fact, shared mechanisms between the two create a vicious cycle wherein metabolism and mood are intertwined. Therefore, a better understanding of the metabolism–mood interface has become a research priority that is crucial for improving the clinical outcomes for individuals with the combined depression–obesity phenotype.

Moreover, conceptualising depression as an immunometabolic disorder facilitates the development of new treatment strategies that go beyond conventional neurotransmitter-based strategies [25]. Experimental approaches focused on inflammation and metabolism (e.g., recombinant adipokines, adipokine receptor agonists/antagonists, or microbiota-based interventions) have shown promising results in preclinical models [26,27,28,29]. Integrating these strategies with conventional antidepressants could provide combined benefits, especially for patients with immunometabolic features.

In this context, the scope of this narrative review was to examine the multidimensional relationship between adipose tissue and depressive disorder by analysing current evidence in several intertwined domains that may fuel the depression–obesity phenotype. Special focus was placed on the involvement of adipokines in mediating these processes and linking peripheral metabolic signals with central mood regulation. We aimed to outline that the integrated understanding of depression–obesity interactions is important not only for comprehending disease mechanisms, but also for identifying novel therapeutic targets to break the vicious cycle between dysmetabolism and depressive disorder. To further deepen our understanding of this complex crosstalk, more research is needed on the signalling differences in various demographics. This deeper insight would allow future clinicians to personalise the treatment of immunometabolic depression.

## 2. Depression Incidence in Obesity

Defined as eminently clinical, mood disruption with persistent feelings of sadness and loss of interest and pleasure are common psychiatric findings in individuals with depression [30]. According to the World Health Organization’s (WHO) assessment report on the global burden of disease, depression ranked as the second leading cause of disease burden and disability worldwide as of 2020 [31]. Additionally, it is anticipated to become the foremost contributor to the global disease burden by 2030 [32]. Estimates conclude that depression afflicts globally over 350 million people from all social backgrounds [32], with the highest prevalence being in Europe, at 11.32%, and the lowest in Asia, although that might be an underestimation due to the scarcity of epidemiological data for this region [31,33,34]. These geographical differences reflect the economic, social, and educational background influences on the genesis of depression [33,34,35].

The past two decades have seen the incidence of depression doubling, especially in low- and middle-income developing countries [34]. This surge can be due to the higher associations between depression and other pathologies, which can range from 9.3% to 23% [33,34,36]. In this regard, angina, arthritis, asthma, diabetes, and obesity are amongst the somatic disorders that co-occur most frequently with depression [33]. Obesity may be an independent predictor of depression [37]. Obese individuals have a higher probability of experiencing moderate to severe depressive symptoms (53.3%) compared to their non-obese counterparts (26.0%) [37]. Regarding obesity–metabolic disorder comorbidities (e.g., hypertension, dyslipidaemia, and hyperglycaemia), the prevalence of depression increases with the degree of metabolic dysfunction regardless of age or gender [38]. Depression’s prevalence is higher (18.8%) in obese metabolically unhealthy individuals vs. obese metabolically healthy patients (17.1%) [38]. This suggests that metabolic health strongly influences depressive risk and severity of symptoms [37,38]. This is likely because of some shared mechanisms (systemic inflammation, neuroendocrine dysregulation) and the consequences on functional capacity and psychosocial stress [10,33].

The first symptoms of depression can be generally identified even in early life, although the median age at onset ranges between 30 and 35 years [16,17]. The prevalence of depressive disorder in children between ages 3 and 11 is rather low, varying from 0.08% to 1.7% [39]. The transition from childhood to adolescence is accompanied by an increase in rates of depression [39]. Thus, a prevalence between 5.6% and 20% was reported in adolescents, with a 12-month incidence of 3.3% [39].

Although there is a trend for earlier onset in males, the reported overall incidence in females is higher; thus, having a depressed mother has emerged as a potential risk factor [1,40]. This gender disparity especially manifests after puberty, without any significance in early ages [39]. However, there are no gender-related differences in remission and recurrence rates, which are important indicators in the epidemiology of depression [1,40]. Rather than being the exception, recurrence is a constant feature, considering that at least 18% of patients endure ten ore more episodes and over 75% of patients have at least one more episode in their lifetime [1,2].

A significant, bidirectional relationship between depression and obesity has also been documented. Obesity is a disorder of energy homeostasis, defined as a body mass index of 30 kg per square metre or above [41,42]. It affects over one billion people worldwide, approximately 13% of the global population, including 650 million adults, 340 million adolescents, and 39 million children [41,42]. Since 1975, the number of obese individuals has tripled, and there seems to be only an upwards trend [41]. In this regard, the global prevalence of obesity is expected to rise to 1.12 billion people by 2030 [43]. Mendelian randomisation studies have shown that increased adiposity produces an increased risk of depressive disorder by almost two-thirds, whereas depression renders individuals more susceptible to gaining weight [14,15]. In fact, 43% of depressed adults match the criteria for obesity [14,15]. Additionally, a meta-analysis revealed that obese patients had a 55% higher risk of developing depression, while those with depression had a 58% elevated risk of developing obesity [44]. Moreover, a 32% higher risk of developing depression has been registered in obese patients compared with those with normal body weight [10]. However, no significant association between depressive symptoms and obesity phenotypes has been reported in the elderly population [45]. Regarding gender disparities, the incidence of depression is roughly double in non-morbidly obese adult females compared to non-morbidly obese adult males [46]. This striking difference attenuates in patients with a BMI of over 40 kg/m^2^ [46].

Frequently, depression and obesity co-occur in youth, though estimates vary by study design and population, with a 2017 meta-analysis finding that patients with obesity had 34% higher odds of having depression [47]. Childhood obesity and depression share numerous risk factors, including sedentary behaviour, unhealthy diets or family environment, and psychosocial stressors [48]. The depression–obesity link in youth appears stronger in girls than in boys and in non-Western populations, probably because the cultural norms and stigma around weight impose greater psychological burden on girls in many societies [47,48]. However, a clear causality between both disorders in childhood has not been yet fully established, with multiple studies reporting mixed results [49].

Pregnancy is a unique life stage where both depression and obesity have significant implications for mother and child [50,51]. Globally, the prevalence of antenatal (prepartum) depression is estimated at around 10–15%, and postpartum depression at around 10–20%, though rates may vary by country and screening methods [50]. At the same time, obesity among pregnant women has become more common, considering that, in many high-income regions, over half of women now enter pregnancy as overweight or obese [51]. Hormonal fluctuation is the main factor in developing perinatal depression [52,53].

Considering all these data, the complexity of the relationship between depression and obesity remains a significant area of research due to its bidirectional nature and its impact on public health.

## 3. Brain–Adipose Tissue–Gut Axis in Depression and Obesity

### 3.1. HPA Axis and Adipose Tissue Interactions in Depression and Obesity

An important contributor to the depression–obesity phenotype is the dysregulation of the hypothalamic–pituitary–adrenal (HPA) axis [11]. A first step in this direction is made through psychosocial chronic stress exposure leading to increased cortisol synthesis [11]. Accordingly, in depressive disorder, approximately 40–60% of all patients present a hyperactive HPA axis. This is demonstrated by increased cortisol levels and failure to reduce cortisol secretion after a dexamethasone suppression test [10,11]. Consequently, this persistent state of hypercortisolaemia promotes the development of central obesity and insulin resistance [11]. If this condition continues, it will decrease the sensitivity of the glucocorticoid receptor (GR), creating an environment in which, despite high cortisol levels, the effects of the hormone are diminished [4]. However, this also impairs the negative feedback loop of the HPA axis, with GRs in the hypothalamus failing to respond to increased circulating cortisol [4].

Hypercortisolaemia also promotes serotonin deficiency and reduces the production of brain-derived neurotrophic factor (BDNF), essential for hippocampal neurogenesis and depression development [3,5,54]. In this regard, HPA axis hyperactivation induces epigenetic modification of neuronal BDNF transcription, either by methylation of BDNF promoters I and IV or by GRs binding to regulatory regions of BDNF exon IV [55,56,57]. Additionally, excess cortisol blunts ERK signalling through the induction of DUSP1/MKP-1 phosphatase that decreases the CREB pathway necessary for BDNF expression and supresses Shp2-TrkB interaction, effectively inhibiting TrkB signalling [58,59,60,61]. Moreover, inflammatory brain events deplete the synapses of dopamine, further activating the HPA axis and disrupting neurogenesis, in chronic stress conditions [3,5,54].

Genetic influences can also increase susceptibility to HPA axis dysregulation. For instance, polymorphisms of FKBP5 genes, a GR activity modulator, determine a disproportionate HPA axis response to stress [62]. This ultimately yields suboptimal weight-loss outcomes under stressful conditions [62]. Another example of how genomic differences impact the HPA axis is the NR3C1 gene, involved in regulating GR sensitivity [63]. Hypermethylation of the NR3C1 gene, commonly seen in patients who have experienced early-life trauma (ELT), has been associated with increased HPA axis activity and increased symptom severity in depressed patients [64]. However, the reverse situation happens in obese patients, in an attempt to regulate HPA homeostasis by enhancing GR sensitivity [63,64].

Evidence shows that more inflammation in depressed patients is associated with increased HPA axis activity and worse therapeutic outcomes [4]. At the same time, adipocyte-secreted cytokines directly impair the central component of this axis, rendering the negative feedback loop inefficient [4]. In this respect, the increased secretion of IL-6 and TNF-α in chronic inflammation-related obesity may activate the HPA axis and promote depressive behaviour [4]. Furthermore, IL-6 and TNF-α may induce microglial activation, which contributes to brain tissue inflammation [65]. Moreover, these adipose tissue-derived pro-inflammatory cytokines are able to promote the indoleamine 2,3-dioxygenase (IDO) activity, which supports the nervous tissue inflammatory status by increasing synthesis of neurotoxic tryptophan catabolites, such as 3-hydroxykynurenine, kynurenine, or quinolinic acid [10,66]. The disruption of the kynurenine pathways has both mood and metabolic consequences for the individuals [4,10,66]. On one hand, it depletes the brain of the main mood-related neurotransmitter (i.e., serotonin) and, on the other hand, of nicotinamide adenine dinucleotide (NAD), which is essential for neural energy metabolism [10]. Therefore, it has been suggested that depression may be associated with the increased secretion of these neurotoxic products and serotonin depletion [4].

The proper functioning of the HPA axis also requires an intact gut microbiota. Amplified stress responses were registered in germ-free mice [67]. Normalisation of the stress response was observed upon colonisation with commensal bacteria [67]. However, the antidepressant potential of the gut microbiota is disturbed by increased cortisol that induces changes in bacterial motility, immunomodulatory activity, and barrier function in depression [6].

**Figure 1:**
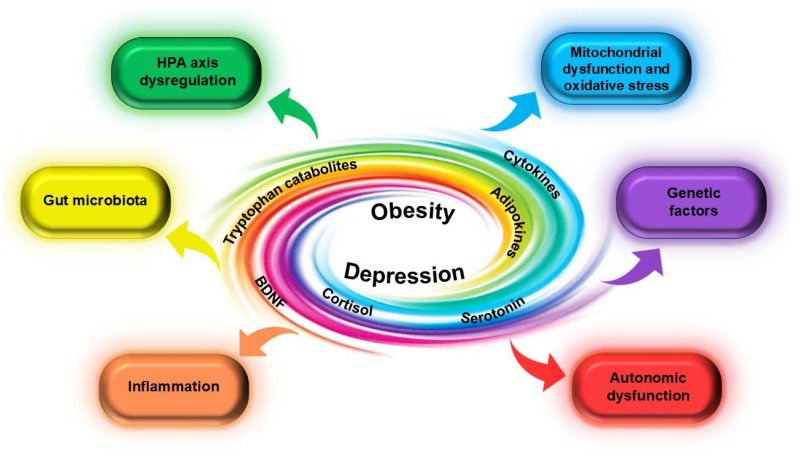
The main mechanisms linking depression and obesity include dysregulation of the hypothalamic–pituitary–adrenal (HPA) axis, genetic factors, gut microbiota dysregulation, autonomic and mitochondrial dysfunction, and a chronic inflammatory profile. Adipokines, cytokines, cortisol, serotonin, BDNF, and tryptophan catabolites play a central role in the pathophysiological mechanisms underlying the depression and obesity phenotype. BDNF—brain-derived neurotrophic factor; HPA axis—hypothalamic–pituitary–adrenal axis.

Obesity-related and depression-related dysbiosis converge into two main points: inflammatory changes and HPA axis dysregulation [6,22] (Figure 1). The dysregulated microbiota of obese people releases lipopolysaccharides (LPSs) into mainstream circulation that alter the brain’s chemistry by reducing serotonin production and blunting dopamine response [6,68]. The neurochemical imbalance is further accentuated by the increased cortisol that can be detected in both pathologies, thus favouring both visceral fat accumulation and mood symptoms [7,9]. Therefore, loss of bacterial diversity and evenness is not enough to explain the pathophysiological changes that lead to either disorder but should instead be regarded as part of a more complex mechanism [69].

Another mechanism of the HPA axis’ involvement in obesity is favouring the expression of orexigenic neuropeptides, i.e., neuropeptide Y (NPY) [70,71]. In turn, increased NPY promotes adipogenesis and angiogenesis in the adipose tissue [72,73]. Moreover, chronic activation of the NPY-NPY2R system increases the odds of developing both obesity and metabolic syndrome [74]. At a neurobiological level, NPY engages the mesolimbic dopaminergic reward system, which drives the individual to select foods that are calorie-dense and rich in carbohydrates [75,76,77]. In this case, NPY acts either within dopaminergic neurons in the ventral tegmental area (VTA) or in the nucleus accumbens (NAc) [78,79,80]. In the former region, NPY activates G-protein-coupled inwardly rectifying potassium currents in order to regulate dopamine neural output, while increasing feeding motivation via Y1 receptors [80]. At the same time, through the same Y1 receptors, NPY directly facilitates reward signalling in NAc, leading to increased fat-rich food intake and reduced striatal neuronal activity [78,79]. Thus, NPY activity in NAc is connected not only to appetite-related dopaminergic functions, but also to mood regulation [78,79,80]. As a consequence, the anorexigenic counteracting effects of corticotropin-releasing (CRH) hormone are blunted by the maladaptive changes that appear as the hyperactivity of the HPA axis is prolonged [81,82].

Considering the accumulated data, HPA axis hyperactivity is a strong connector between depression and obesity, which also strongly influences the clinical and therapeutic course of patients with this combined phenotype [4,11]. Not only do these individuals tend to have quicker metabolic deterioration, but they often exhibit a poorer antidepressant response compared to those afflicted only by depression [83]. Therefore, the need to treat both conditions simultaneously arises in an effort to ameliorate the effects of a dysregulated HPA axis [18]. The restoration of HPA axis homeostasis through pharmacological and non-pharmacological measures might break its vicious cycle, linking these two disorders [4,18,19].

### 3.2. Genetic Factors Linking Depression and Obesity

From a genetic standpoint, approximately a tenth or more of depression’s genetic background overlaps with that of obesity, and multiple specific genes gave been identified as contributors to both conditions [84]. These include genes involved in energy homeostasis, neurotransmission, endocrine signalling, and neural architecture that, by increasing the risk of both disorders, converge to support the combined depression–obesity phenotype [85].

The energy homeostasis category includes genes that are crucial for both mood and metabolic pathways. Their disturbances might affect neurotransmission and neuroplasticity, thus being instrumental in the emergence of both obesity and depression [84,85,86]. This is the case for the fat mass and obesity-associated (FTO) gene, one of the biggest genetic contributors to body mass index (BMI) [86]. FTO encodes a m6A demethylase involved in the epigenetic regulation of some energy-related genes [86]. In the adipocyte progenitor state, FTO-encoded proteins upregulate Iroquois homeobox gene (IRX) 3 and IRX5, while restraining uncoupling protein 1 (UCP1) activity, in order to allow a phenotypic shift in adipocytes, from thermogenetic beige cells to energy-storing white cells [87,88]. When the adipocytes mature, FTO acts as a promoter of adipogenesis by enhancing peroxisome proliferator-activated receptor gamma (PPARG) activity, elevating its effects on fat accumulation [89]. In the hypothalamus, neuronal FTO stimulates axonal trafficking and secretion of appetite-stimulating peptides, such as NPY and Agouti-related protein (AgRP) [90]. Some of its genetic variants, most notably rs9939609 and rs1421085, are associated with depressive risk, illustrating the extension of the bidirectional relationship between depression and obesity into the genetics of these diseases [86,88,91,92]. The rs9939609 variant has enhanced BMI-increasing effects compared to the normal variant, which were found to be further marked in depressed individuals [92]. The rs1421085 variant is directly linked to neuroticism, a personality risk factor for depression, as well as hyperphagia, thus further enabling this gene variant to heighten the depressive risk of an individual [88,91].

Similarly, variants of the neuropeptide Y receptor, NPY2R, have been linked to comorbid depression and obesity [86]. NPY2R’s rs6857715 variant associates not only with more severe obesity, but also with atypical depression [93,94,95]. The connection between atypical depression and this variant might be due to its ability to interfere with peptide YY (a NPY2R ligand) function, which fails to signal satiety [95]. Other NPY2R variants, such as rs12649641, rs2342676, and rs6857530, have additive effects with other significant obesity–depression genes (e.g., FTO and MC4R) [74,96].

Amongst other genes that link depression with obesity, melanin-concentrating hormone receptor 2 (MCHR2), proprotein convertase subtilisin/kexin type 9 (PCSK9), and apolipoprotein A5 (APOA5) may be mentioned, as genes involved in energy-related processes [86]. MCHR2 CC or CT polymorphism is associated with higher BMI in individuals with either a past or present atypical depression diagnosis [97]. This is in part due to the effects of receptor stimulation, namely driving the intake of energy-dense food and increasing anxious behaviour [98]. Although some studies suggest that PCSK9 and APOA5 are positively associated with comorbid depression and obesity, reproducible associations with depressive symptoms are limited, given their strong metabolic effects [86,99,100].

On the other hand, the MC4R (melanocortin-4 receptor) gene, which codes an important protein for appetite suppression, has variants that paradoxically increase obesity risk but have independent effects on depression [86]. Faulty MC4R signalling in the dorsal raphe nucleus interferes with both feeding and anxious behaviour, creating a point that ties obesity and depression in mice models [101]. The rs17782313 variant is associated with emotional eating and food cravings, known attributes of atypical depression [102]. This variant is reckoned to be overexpressed in depressed individuals [102]. The rs17782313 variant’s link to BMI is explained through feeding behaviour alterations rather than by depressed mood, which is independent of weight gain in individuals exhibiting this variant [86,102].

Genes that influence neurotransmitters systems are also essential for demonstrating the shared biology of the depression–obesity combined phenotype. In this context, variants in the monoamine-degrading enzymes, catechol-O-methyltransferase (COMT) and monoamine oxidase A (MAOA), modulate brain dopamine, norepinephrine, and serotonin levels and functions and have been linked to both depression and obesity [86]. These alleles raise depression risk and, in several cohorts, co-occur with binge-eating and higher BMI [86]. In this respect, among adolescents, MAOA variants predicted depressive symptoms and interacted with BMI status [103]. Other components of dopaminergic signalling, such as the dopamine transporter 1 (DAT1), encoded by solute carrier family 6-member 3 (SLC6A3) gene and receptors, like dopamine receptor 4 (DRD4), may connect anhedonia with overeating and subsequent weight gain [104]. Similarly, serotonin-pathway genes connect depression and obesity: loss-of-function mutations in the appetite-regulating receptor gene HTR2C predispose the individual to hyperphagia and severe obesity, while other serotonergic variants, such as HTR2A polymorphisms, have been associated with postpartum depression [105,106].

Disrupted control of the HPA axis may additionally explain why depression and weight gain often coexist. Risk alleles in GR gene NR3C1 keep cortisol elevated, promoting visceral adiposity and stress-induced mood episodes [86,107]. Polymorphisms in CRHR1 boost depression risk after ELT and, in mice, alter feeding patterns [107,108]. Meanwhile, alleles in the steroid-metabolising gene, AKR1C2 (aldo-keto reductase-1 C2), are overexpressed when obesity and depression overlap, indicating disturbed neurosteroid signalling [86]. The overexpressed gene alters allopregnanolone metabolism, a BBB-crossing molecule that interacts with γ-aminobutyric acid (GABA) signalling, thereby contributing to depressed mood in obese people [109,110].

Large-scale omics point out neuronal growth regulator 1 (NEGR1) as a main genetic link between obesity and depression [111]. Initially tied to body weight, it now shows genome-wide association with depressive risk as well [85]. NEGR1 supports neuron growth and synaptic flexibility in feeding–reward circuits, while its expression is reduced in the hypothalamus of obese individuals and in the prefrontal cortex (PFC) of patients with depression [85,112]. In rats, NEGR1 loss reduces reward responses and heightens stimulant sensitivity [85,113]. Because both disorders feature reward-circuit deficits (anhedonia and compulsive eating), NEGR1’s impact on limbic pathways might support the combined phenotype [114].

However, this genetic background is also impacted by environmental and psychosocial factors. The BMI augmentation potential of the FTO risk allele can be either attenuated by physical exercise or heightened by a high-calorie diet or a sedentary lifestyle [86,115]. Ultimately, the FTO–environment interaction leads to depression when it is accompanied by lowered self-esteem, social stigmatisation, or metabolic changes [86,115]. Along the same lines, carriers of the serotonin transporter promoter short allele (5-HTTLPR in SLC6A4) do not develop depression, unless exposed to significant chronic stress [105]. Also, variants of the HPA-related genes (CRHR1, NR3C1) possess a double threat, since their presence allows for increased cortisol in response to stress and, therefore, increased central weight gain and depressed behaviour [107,108].

Notwithstanding, the genetic background of an individual can influence their environmental exposure. Several studies have used polygenic risk scores (PRSs) to measure these gene–environment interactions [116,117,118]. Patients with a higher PRS for BMI were more prone to psychosocial stress in early life (e.g., adversities and bullying) due to health issues or weight-related stigma, which in turn predicted more probable depressive symptoms [116]. Conversely, a higher PRS for depression is more probable to lead to that individual developing depressive disorder if they also had a higher BMI compared to normal-weight individuals [117]. At the same time, certain depressive behaviours (emotional eating or physical inactivity) may allow an obesogenic genetic background to fully manifest [118].

Not least, gender modifies the relationship between depression and obesity, with women carrying twice the risk of depression and higher obesity rates after the age of 20 years [119]. Higher BMI implies a stronger effect on depressive risk in females compared to males [120]. Female-specific genetic variants, such as the oestrogen receptor genes and risk alleles such as FTO and MC4R (whose rs17782313 associates with atypical depression only in females) modulate stress and adiposity [104,119,121,122]. Moreover, obesity in pregnant patients increases the odds of antenatal and postpartum depression through a complex genetic crosstalk between polymorphisms of serotonin (HTR2A), catecholamine (COMT and MAOA), HPA-axis (CRHR1), and oestrogen receptor genes [86,105].

The shared polygenic profile of the combined depression–obesity phenotype spans genes involved in energy metabolism, neurotransmission, endocrine signalling, and neuronal function, thus linking adipose tissue to a wide array of mood and feeding brain circuits [54,84,86]. All in all, these genes drive behaviour toward hyperphagia and disrupt reward pathways, while increasing stress sensitivity and adipocytes’ energy-storing capacity [86,88,101]. The complex genetic milieu is amplified by social and lifestyle obesogenic factors, urging a clinical strategy that integrates weight management and risk factor management [86,105,121].

### 3.3. Adipose Tissue-Associated Inflammatory Status Linking Depression and Obesity

Inflammation, especially in its low-grade chronic form, has been postulated as the common pathophysiological denominator between depression and excessive adipose tissue in obesity [10,20]. In obesity, pro-inflammatory adipocytes-derived cytokines (IL-6, TNF-α, and IL-1β) and C-reactive protein (CRP) are often elevated [20]. Similarly, in depression, there is upregulated cytokine production both in CNS and in the adipose tissue [3,5,123,124,125]. These cytokines are seized due to a faulty BBB and exert negative effects on neuroendocrine, monoaminergic, and oxidative stress systems, leading to a hypodopaminergic state, neurotoxic metabolites buildup, and decreased neuroplasticity and neurogenesis. Each particular inflammatory profile outlines a different depression endotype [3,5,123,124,125]. Unsurprisingly, the depressive risk of metabolically unhealthy individuals is notably heightened, in particular in those with persistent increases in IL-6 and TNF-α, which are released by excessive adipose tissue in obese patients [10,20,21]. The inflammatory phenomena in depressive disorder are various and differ from one individual to another. In this regard, the literature recognises seven inflammatory endotypes that can be identified in depression [7,56,126,127,128,129,130,131,132,133,134,135,136,137,138,139], although research in the inflammatory profiling of depressive disorder is still underway (Table 1). These endotypes can be differentiated based on specific biomarkers and certain clinical features that are more prevalent within a certain endotype. The inflammatory profiles overlap to a certain extent with the inflammatory environment of obesity. Chronic low-grade inflammation, translated as increased IL-6, TNF-α, and hsCRP, is present in both obesity and in several endotypes of depression (immunometabolic depression, inflammation-related dopaminergic dysfunction, early-life-stress inflammatory profile, and low-grade inflammatory depression) [71,126,127]. At the same time, the adaptative immunity in individuals with excess adipose tissue mass shifts towards a Th1/Th17 pro-inflammatory profile that is congruent with the Th17-oriented profile of depression [140].

Obesity and immunometabolic depression also share metabolic disturbances (e.g., increased leptin and insulin and reduced adiponectin). These conditions are interconnected, with obesity-induced metabolic dysfunction and chronic inflammatory status impacting brain function and mood regulation, leading to an increased risk for depression [127,128,141,142]. It is important to note that gliosis could also be present in obesity, especially within the hypothalamus [143]. Gliosis involves astrocytes and microglia, and it was correlated with the neuro-inflammatory status and metabolic disturbance that occur in obese patients. This chronic inflammatory status may trigger gliosis, suggesting a shared neuroinflammatory mechanism linking obesity and depression [129,130,131].

Moreover, there is evidence supporting the infiltration of the excess adipose tissue in obese patients with M1 pro-inflammatory macrophages, which secrete IL-1β, IL-6, and TNF-α [10,144]. Upon secretion in the bloodstream, these cytokines interact with toll-like receptor-4 (TLR-4), nuclear-factor-κB, (NFκB), and NKRP3-inflammasone in various organs, including CNS [145,146]. NF-κB is further activated by endocrine disruptions that are created by excess white adipose tissue, namely the high leptin-to-adiponectin ratio [144,147,148]. At the same time, the continuous stimulation of IL-6 and TNF-α signalling cascades hyperactivate the HPA axis. In turn, this leads to increased cortisol and adrenocorticotropic hormone (ACTH) levels and the consequent desensitisation of GRs on immune cells, resulting in persistent cytokine secretion [11,149,150].

Increased adiposity also favours a pro-inflammatory adaptative response expressed as a reduction in regulatory T lymphocytes (Treg) and an increase in CD8+ T cells [140]. CD8+ T cells infiltrate obese adipose tissue to facilitate macrophagic recruitment and M1 polarisation [151]. Simultaneously, the adipocytes overexpress class II major histocompatibility complex (MHC-II), activating CD4+ lymphocytes to secrete IFN-ɣ [152]. Metabolic signals assist in the genesis of this maladaptive immune response, since hyperleptinaemia drives Th17 polarisation and hypoadiponectinemia removes the barriers against lymphocyte activation [153,154].

Higher peripheral cytokines (IL-1β, IL-6, and TNF-α) also lead to increased BBB permeability, allowing them to reach CNS, alongside with endotoxins, adipokines, and glucocorticoids [7]. In the context of frequently heightened psychosocial stress in depression, microglia are more reactive to inflammatory stimuli [155]. Consequently, these stimuli converge to promote microglial activation [7,155,156]. Supplementary, high-fat diets result in microglial activation within the mediobasal hypothalamus in animal models [156,157]. Additionally, the degranulation of hypothalamic mast cells can further activate the microglia [158]. Once activated, the microglia secrete IL-6 and nitric oxide (NO) and interfere with the function of proopiomelanocortin (POMC) dendrite, as well as affecting leptin and insulin signalling, thereby inducing hyperphagia and weight gain [156,158,159]. Hyperphagia is further maintained by LPS-induced hedonic feeding, in which peripheral LPS enters CNS through a faulty BBB and creates TLR4-dependent neuroinflammation [146,160]. The findings are sustained via imaging studies on animal models that demonstrate enhanced microglial activation in the hypothalamus of obese patients, along with increased IL-6 and microglial CX3CL1 secretion in the cerebrospinal fluid [156,159].

Adipocytes from excessive white visceral adipose tissue have the ability to reduce serotonin synthesis by directing tryptophan metabolism towards kynurenine via IDO-1 overexpression [161,162]. In patients with the combined depression–obesity phenotype, the kynurenine-to-tryptophan ratio was higher than in either disorder separately [162]. The adipose-induced disruption of tryptophan metabolism is mainly due to increased pro-inflammatory cytokine production and secretion (IL-6 and TNF-α) [10]. Kynurenine passes through BBB and is converted into quinolinic acid that has the property of binding to the *N*-methyl-d-aspartate receptor (NMDAR), resulting in glutamate excitotoxicity [161,163]. This is especially important, since elevated levels of glutamate lead to an imbalance of neurotransmission through GABA and glutamate systems and a hypermetabolic state that results in volumetric changes in the grey matter in certain areas [3,5]. The inflammatory microenvironment created by excess adiposity reduces the GABA levels, which, in depressed patients, is already low, thereby impairing emotional processing and cognitive performance [10]. The successful treatment of depressive disorder results in the normalisation of GABA levels and restoration of GABA-ergic system function, which may have beneficial effects on obesity, since this neurohormone exhibits anti-inflammatory effects within the white adipose tissue [10].

Finally, gut dysbiosis has been proven to compromise the barrier function of the intestinal tract, allowing for numerous pro-inflammatory compounds to enter the systemic circulation [6]. In turn, these compounds, especially LPS, activate immune cells to trigger several molecular cascades that exacerbate the inflammatory response through the secretion of cytokines (IL-6 and TNF-α) [164]. At the same time, dysbiosis-induced inflammation might reduce neurotrophic factors, accelerate cognitive decline, and increase the severity of depressive symptoms [165].

Moreover, several pro-inflammatory cytokines that play an essential role in depression, such as TNF-α, IL-1β, and IL-6, are suppressed by ghrelin [10]. Molecularly, ghrelin’s anti-inflammatory effects are due to its ability to inhibit the NF-κB pathway [10,166]. This action has been attributed to vagal nerve stimulation, implicating that the malfunctioning of the ghrelin system is involved in the autonomic dysfunction from depression [167]. Another explanation for the immunomodulatory role of ghrelin in depression comes from probiotic-supplementation studies [166]. Such studies postulate that probiotics lead to a decrease in inflammation and depressive symptoms by restoring ghrelin levels [166].

The importance of gut microbiota in neuroinflammation is also highlighted by its role in the activation and maturation of microglia [168]. In this context, microglia are aberrantly activated, especially in the PFC and the hippocampus, resulting not only in increased number and size, but also in an altered cytokine secretion profile [169,170]. Their activity is further stimulated by the lack of inhibition that microbial metabolites provide, which have the capacity to dull the microglial inflammatory reaction [168,171,172]. These responses were reversed by bacterial recolonisation, thus demonstrating the modulatory role of gut microbiota on the CNS inflammatory response [67].

To summarise, inflammation lies at the interface between depression and obesity. The inflammatory adipose tissue milieu in obesity is mirrored by inflammatory endotypes of depressive disorder, with a higher depressive risk for metabolically unhealthy individuals [10,20,21]. Systemic inflammation converges with HPA axis hyperactivation not only to perpetuate an inflammatory loop, but also to induce neuroinflammation through activated microglia, therefore disturbing both feeding and mood circuitry [7,10,11]. This is further reinforced by the effects of gut microbiota on neurotransmitter’s balance, thereby supporting the argument for an integrated brain–gut–adipocyte axis in obese patients [3,5,67,168].

### 3.4. Gut Microbiota Role in Obesity–Depression Axis

The last decade of biomedical research has unravelled the important role of gut microbiota in human health as a major contributor to immune system development, maintaining intestinal barrier integrity and metabolism [6]. More and more studies pinpoint the implications of commensal dysbiosis on systemic inflammation, metabolism, and brain function, transforming this diverse ecosystem into a nexus point for a variety of human disorders, including depression and obesity [6,7,8].

Several studies illustrate the alterations in gut microbiota composition and diversity in individuals with depression [6,9,67,173]. In this regard, it has been found that depression is associated with a reduction in butyrate-producing bacteria, such as *Firmicutes*, *Bacteroides*, *Coprococcus*, *Faecalibacterium*, and *Subdoligranulum* [9,173]. On the other side of the spectrum, a series of opportunistic or pro-inflammatory bacteria are overrepresented in depressed patients, such as *Lactobacillus*, *Streptococcus*, *Fusobacterium*, *Clostridoides*, and, most notably, *Eggerthella* [9,67,173].

Similarly, the composition and evenness of the bacterial microbiota in obesity is disturbed, as well [22,174]. Bacterial diversity is also reduced, yet, in this case, studies have remarked increased proliferation of fermenting bacteria, such as *Ruminococcus*, *Lactobacillus*, and *Clostridoides*, allowing for more food-extracted energy [174]. The most consistent finding in obese patients is the increased *Firmicutes* (as well as the above-mentioned bacteria) to Bacteroidetes ratio, further confirming the increased calorie absorption in these individuals [174]. As expected, bacteria that are associated with lower BMI (*Akkermansia*, *Bifidobacterium*, and *Christensenellae*) are severely depleted in obese patients [22].

The complex bacterial ecosystem of the gut communicates with the CNS via bacterial metabolites and neurotransmitters. Particularly, butyrate, which has been extensively studied for its antidepressant capacity, is able to cross the BBB [6,9]. In CNS it can inhibit deacetylation of histones to modulate gene expression and regulate neuroimmune responses by G-protein-coupled receptors [6,9]. A factor contributing to this process is butyrate’s ability to enhance vagal tone and cholinergic signalling [9]. Also related to vagal activity, GABA-producing bacteria (some *Lactobacillus* strains) might increase this neurotransmitter’s levels in the brain, therefore alleviating depressive symptoms [7,9]. Finally, the gut is the main source of serotonin in the body through the enterochromaffin cells (ECs) and enteric neurons [175]. In this regard, two hydroxylase (TPH) isoforms are responsible for tryptophan metabolism in the gut, namely TPH1 (predominantly in EC) and TPH2 (in enteric neurons) [176]. THP1 expression is severely diminished in germ-free mice, highlighting the importance of the gut microbiota in tryptophan metabolism regulation. The resulting serotonin is sequestered by platelets and cannot cross the BBB, but it indirectly influences CNS via vagal signalling and the modulation of platelet and immune functioning [175,176]. Furthermore, dysbiosis is thought to shift tryptophan metabolism towards the kynurenine pathway, reducing the production of serotonin and the availability of tryptophan in the CNS [6,68].

As mentioned before, in obesity, there is an overabundance of fermenting bacteria, allowing the host to extract up to 10% more energy out of consumed food [22]. Beyond its role in calorie economy, the metabolites produced by commensal bacteria interact with receptors of enteroendocrine, immune, and neural cells and can induce the secretion of peptide YY, glucagon-like peptide-1 (GLP-1), and ghrelin, thus stimulating appetite and lipogenesis [177,178]. These molecules can also act via vagal circuits to influence hypothalamic centres of appetite and satiety [6,179,180]. Finally, metabolic endotoxemia, expressed as chronically elevated LPS, impairs insulin signalling, having an adiposity-increasing effect and favouring adipose tissue inflammation [6,22].

In the light of these findings, gut microbiota can be considered a nexus point between immunity, metabolism, and brain function, influencing the risk for depression and obesity alike [6,7,8]. Although, as far as diversity is concerned, both disorders stand at opposite ends of the spectrum, the pathways facilitated by bacterial metabolites, the bacterial modulation of tryptophan metabolism, and the microbiota-mediated inflammatory response create a strong link between depression and obesity [7,9,175,176].

### 3.5. Autonomic Dysfunction Linking Obesity and Depression

Involuntary bodily functions (i.e., blood pressure, respiratory rate, digestive tract motility, etc.) are within the control of the autonomic nervous system (ANS), a network of neurons whose axons stretch throughout the body from the spinal cord [181]. It comprises two systems: the sympathetic ANS (“fight-or-flight” type of responses) and the parasympathetic ANS (“rest-and-digest” type of responses) [181]. Physiologically, there is a balance between the functioning of its branches, which seems to be disrupted in both depression and obesity, as shown by an ever-increasing body of evidence [4,182,183,184].

In depression, the vagal tone, as measured by heart rate variability (HRV), is significantly diminished, illustrating the dominance of the sympathetic ANS [183,185]. This effect on HRV is still present in depressed patients that are not under any treatment, as demonstrated by a 2016 meta-analysis [183]. This signifies that this ANS imbalance is a result of depression itself and not of the treatment [183]. Additionally, sympathetic overactivation in depressed patients is expressed as elevated heart rates and increased cardiac outflow. The heightening of these cardiac markers is directly proportional to the severity of depressive symptoms [182,186]. Therefore, not only is depression a cause for autonomic dysfunction, but it is also a recognised risk factor for cardiovascular disease, with ANS measurements serving as possible biomarkers for both depressive and cardiovascular disorders [185,187].

On the other hand, there is a bidirectional relationship between metabolic disorder and autonomic dysfunction in obesity [184]. The initial sympathetic dominance directly impacts energy homeostasis by reducing satiety and energy consumption [182,184]. These changes promote weight gain, which ultimately leads to obesity, whose natural course involves autonomic complications, such as arterial hypertension, tachycardia, or restrictive ventilatory dysfunction [184]. In turn, these complications are a sign of vagal withdrawal and sympathetic dominance, low HRV reflecting the latter [188,189]. Interestingly, autonomic dysfunction in obesity targets the vessels of the kidneys and skeletal muscles, suggesting that the autonomic complications of obesity stem from visceral dysregulation [182].

Both depression and obesity exhibit autonomic dysfunction, expressed as sympathetic ANS dominance, albeit targeting different organs (the cardiovascular system in depression, the kidneys and skeletal muscle vasculature in obesity) [4,182]. Parasympathetic withdrawal in obesity may act as an independent risk factor for depression, which, in turn, increases stress response and predisposes to unhealthy eating behaviours [182].

Chronic stress might be the common denominator between the autonomic dysfunction in both disorders [4]. Long-term exposure to stress activates both the HPA and the sympathetic ANS, increasing the central fat accumulation and aggravating mood symptoms [4,182].

To conclude, the common pattern of autonomic dysfunction highlights the common pathophysiological interrelationship between obesity and depression [183,188]. The sympathetic ANS dominates both disorders through different organ targets in each, whereas the parasympathetic vagal tone is significantly diminished [4,182]. Chronic stress is one of the elements that can fuel autonomic dysfunction in these disorders, emerging once again as a bonding agent between their pathogenic mechanisms [4,182,185].

### 3.6. Mitochondrial Dysfunction and Oxidative Stress in Depression–Obesity Phenotype

During the last years, mitochondrial dysfunction gathered recognition as a contributor to the pathogenesis of numerous pathologies [11]. Mitochondria are organelles that govern adenosine triphosphate (ATP) production and regulate several intracellular processes, like the generation of reactive oxygen species (ROS), calcium signalling, and apoptosis. Both adipose and nervous tissue are highly dependent on efficient mitochondrial function; therefore, their malfunctioning is expected to be involved in the biology of both disorders [11,12].

From a microscopic point of view, mitochondria of depressed subjects present fewer mitochondrial cristae, signifying disturbed electron transport chain functioning and, consequently, reduced ATP formation [12,190]. This is congruent with magnetic resonance spectroscopy studies that have shown a decreased level of phosphate metabolites in the brain of depressed patients [191]. This could have several explanations, including a reduction in the activity of enzymes comprising complexes I, II, and III or the downregulated expression of genes modulating oxidative stress and ATP synthesis [12,192]. Moreover, some experimental mitochondria-targeted compounds produce antidepressant effects in addition to their upregulation of mitochondrial biogenesis, further advocating for the role of mitochondrial dysfunction in depression [193].

Neuroplasticity is severely dysfunctional in depressive disorder, with hippocampal atrophy and reduced neurogenesis [194]. Since all processes related to neuroplasticity and synaptic neurotransmission require a tremendous amount of energy, it is thought that impaired mitochondrial function could be one of the underlying causes of these deficits [12,190]. In this regard, the mitochondria’s role in mediating apoptosis should not be overlooked, considering that stress-induced neural apoptosis has been associated with elevated cytochrome-c release and caspase activation [195]. Furthermore, physiological effects of chronic stress, such as the activation of the HPA axis, additionally aggravate mitochondrial defects by reducing their bioenergetic capacity and altering mitochondrial gene transcription [190].

Oxidative stress offers not only possible pathophysiological explanations for depressive disorder but also represents a valuable marker for disease severity and prognosis. Depressed patients present with a reduction in key antioxidant enzymes, such as superoxide dismutase and glutathione peroxidase, thus favouring oxidative damage of the nervous tissue and mitochondrial genetic material via ROS [10,12]. This creates a vicious cycle that accentuates mitochondrial dysfunction and impairs neuroplasticity and neurotransmission [190]. Some antidepressants (e.g., fluoxetine, venlafaxine, trazodone, etc.) have been reported to break this cycle by modulating the expression and activity of such enzymes [196].

In obesity, the excess nutritional load might overwhelm the oxidative capacity of the mitochondria [197]. Firstly, adipocytes of obese patients exhibit fewer mitochondria with more structural abnormalities (smaller-sized and abnormal cristae), which signifies lower oxidative ability, including for fatty acids oxidations, allowing for the enlargement of cells’ fat deposits [198,199]. Secondly, these adipocytes present heightened levels of mitochondrial ROS that not only impair adipogenesis and thermogenesis but also interfere with the normal differentiation of the adipose cells, facilitating the formation of hypertrophic, insulin-resistant phenotypes [198,199]. Additionally, the pro-inflammatory cytokine-rich environment of the obese patients’ adipose tissue, especially when TNF-α levels are increased, is conducive to mitochondrial fragmentation and cristae loss [199].

Pro-inflammatory cytokines secreted by the adipose tissue in obese patients can cross the BBB and induce neuroinflammation and oxidative damage, particularly in the hypothalamus and the hippocampus [11,200]. In either disorder, nuclear factor erythroid 2-related factor (Nrf2) antioxidant activity is diminished, making the tissues more susceptible to the oxidative processes. This results in a supplementary increase in the inflammatory load on both tissues, providing that reduced Nrf2 activity also removes its inhibition over the NF-kB pathway, which is already overstimulated in both pathologies [10,200].

Considering the metabolic abnormalities of obesity (leptin resistance and hypoadiponectinemia) that accentuate both inflammation and oxidative damage, mitochondrial dysfunction can be considered a central point in the biology of immunometabolic depression [127]. Systemic inflammation and oxidative damage associated with obesity can compromise brain mitochondria, whereas the neuroendocrine consequences of depression can impair metabolic control [10,11,12]. However, more research is needed to understand the role of mitochondria in the development of the combined depression–obesity phenotype.

### 3.7. Integrative Summary: The Brain–Adipose Tissue–Gut Axis in Depression and Obesity

The interaction between the CNS, adipose tissue, and the gut creates a dynamic regulatory triad, crucial for both metabolic and affective homeostasis. This complex circuit is supported by the constant dialogue between neuroendocrine signalling, inflammatory pathways, and microbial activity [3,4,19,67,175]. This triad regulates systemic metabolic function and enables the brain–metabolism link [5,6,7,10,176].

The HPA axis is activated by chronic psychosocial stress and metabolic dysfunction, leading to persistent glucocorticoid release and sympathetic overdrive. These effects impair hippocampal neuroplasticity and promote visceral adiposity and chronic inflammation, associated with increased TNF-α and IL-6 [5,6,7,10,176].

Adipose tissue acts as an endocrine mediator of brain function by secreting various adipokines that control appetite, reward processing, and mood [10,11,67]. Dysfunction of adipose tissue causes systemic inflammation and increased ROS. In turn, they contribute to microglial activation and astrocytic reactivity [6,7,175]. Thereby, dysfunctional adipose tissue interferes with neurotransmitter balance and synaptic plasticity.

The gut microbiota is a central mediator of this triad, as alteration in microbial composition disturbs both mood and metabolism. This is possible due to the disturbed microbial metabolite balance that hinders serotonin synthesis and microglial regulation [6,7,67]. Furthermore, dysbiosis further reinforces depressive behaviour by activating the HPA axis and promoting systemic inflammation via increased intestinal permeability [6,9,11].

Collectively, the components of this axis create a self-sustained loop that might explain the depression–obesity phenotype and the limited efficacy of interventions targeting either depression or metabolism [4,11,103]. Understanding the mechanisms of this axis allows for an integrative approach of this phenotype and the development of multimodal approaches [6,7,9,18]. Interventions aimed at restoring HPA axis homeostasis, normalising gut microbiota, and involving adipokine-based therapeutics might be the key to the efficient treatment of this comorbidity. The main studies exploring the brain–adipose tissue–gut axis in depression and obesity are illustrated in Table 2.

## 4. Adipokine–Brain Interactions in Depression

Adipose tissue is more than an energy storage compartment; its capacity to secrete over 600 adipose-derived hormonal factors defines it as an endocrine organ. Therefore, adipose tissue is able to regulate a myriad of bodily processes, including metabolism, immunity, and behaviour [201].

Obesity is a chronic disease characterised by excess adipose tissue, accompanied by a state of chronic inflammation and dysregulation of adipokines balance. This may lead to obesity-related pathologies, such as cancer, diabetes mellitus, and chronic cardiac and renal diseases [13,201,202,203]. Visceral adipose tissue has been recognised as a key player in cardiovascular and metabolic health [204,205]. Amongst its compartments, epicardial adipose tissue represents a measurable marker for certain dysmetabolic features [204,205]. Multimodal imaging allows clinicians to assess visceral adipose tissue both structurally and metabolically, guiding precise prevention strategies. The data provided by imaging methods directs early anti-inflammatory, antihyperglycemic, and lipid-lowering therapies [204,205].

Adipokines can cross the BBB, exerting their effects upon the CNS [13]. In obese patients, adipokines promote neuroinflammation and elicit a heightened stress-response of the HPA axis [10]. Moreover, adipokines are part of the molecular mechanism that prompts neuroplasticity and synaptic efficacy in key areas involved in mood and emotional processing, the hypothalamus and the hippocampus [10,13]. Not least, adipokines ensure the functioning of neuronal metabolism, which is necessary to sustain not only the survival of the neuron, but also its involvement in processes that require a significant amount of energy [10,13]. Therefore, adipokines are implicated in the immunometabolic pathways that define depression’s neurobiology, its imbalance predisposing the individual to this disorder.

Key adipokines involved in the pathophysiology of depressive disorder include leptin, adiponectin, apelin, resistin, chemerin, omentin, visfatin, and pro-inflammatory cytokines (IL-1β, IL-6, and TNF-α), the latter being previously described.

### 4.1. Leptin

Leptin is among the best-characterised adipokines, and circulates in the blood in a concentration that is proportional to adipose tissue expansion [142]. It exerts its functions via the leptin receptors (LEP-Rs) that are found throughout the body, including in the nervous tissue [13,142]. Within the CNS, LEP-Rs are also expressed by neurons and glial cells [206,207]. Microglia and astrocytes that express this receptor are involved in a variety of processes, including the modulation of synaptic output and synaptic plasticity [206,207]. The long isoform Ob-Rb is the major signalling LEP-R in the nervous tissue, while the remaining LEP-Rs isoforms (short isoforms) are involved in leptin transport or availability in CSF, but do not trigger the complete intracellular signalling pathway [206,207,208].

In the CNS, leptin acts primarily as a regulator of the hypothalamic satiety centre, in the arcuate nucleus [13]. However, the expression of LEP-Rs is not restricted to this region, as they can be found in several other brain regions involved in mood regulation, such as the hippocampus, amygdala, VTA, and PFC [13,209].

**Figure 2:**
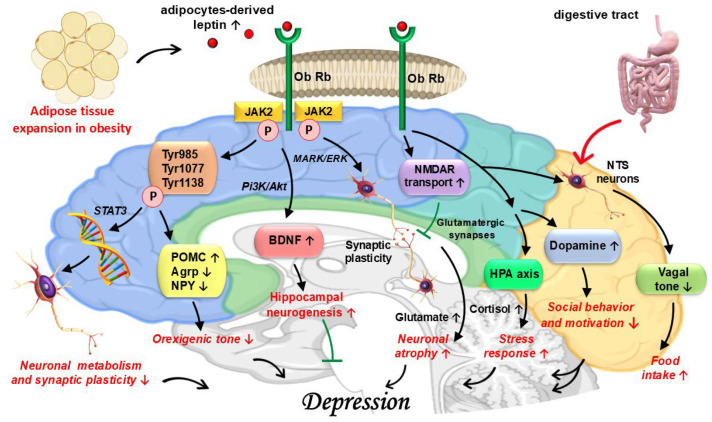
Potential mechanisms of leptin in the neurobiology of depression in obesity. Agrp—Agouti-related protein; BDNF—brain-derived neurotrophic factor; HPA axis—hypothalamic–pituitary–adrenal; JAK2—Janus kinase 2; NMDAR—*N*-methyl-d-aspartate receptor; NTS—*nucleus tractus solitarius*; NPY—neuropeptide Y; Ob Rb—Ob-receptor b isoform; Pi3K/Akt—phosphatidylinositol 3-kinase/protein kinase B; POMC—proopiomelanocortin; STAT3—signal transducer and activator of transcription 3; Tyr—tyrosines; ↑—increase; ↓—decrease; →—activation; ꓕ—inhibition.

Leptin is involved in the neurobiology of depression through several mechanisms, including complex intracellular signalling cascades, NMDAR regulation, modulation of BDNF expression, and brain reward circuitry functioning [210,211,212]. These pathways allow leptin to become a potent actor not only of neuronal metabolism, but also of neuronal and synaptic plasticity [210,211]. The ligand–receptor bond launches one of the following intracellular signalling cascades: JAK/STAT3, PI3K/Akt, or MAPK/ERK [206,210,211]. The first cascade involves two phosphorylation steps (JAK2 autophosphorylation and the phosphorylation of three intracellular tyrosines, Tyr985, Tyr1077, and Tyr1138, which ultimately activate STAT3), allowing its final product to reach the nucleus, where it regulates the transcription of genes involved in neuronal function and metabolism [206,211] (Figure 2). Most importantly, in the hypothalamus, this pathway increases POMC transcription, while repressing Agrp and NPY, proving leptin’s central role in the control of the orexigenic tone [213]. The last two cascades are critical regulators of leptin’s effects on food intake and energy balance and can regulate neuronal growth, survival, and synaptic plasticity, depending on the downstream substrates that they modulate [211,214].

Leptin plays a significant role in counteracting the usual neuronal atrophy that is found in depression [210,213,215]. This is possible by influencing the expression of BDNF via the PI3K/Akt pathway [212]. Leptin increases the histone acetylation of the BDNF promoters, thus increasing the transcription of its gene, the latter process being deficient in depression [212]. Through ERK-mediated mechanisms, leptin is also able to influence the synaptic plasticity of the reward and cognitive circuitry. This effect is complementary to leptin’s action on NMDAR, mechanisms that are to be discussed later [216].

The glutamate system is intricately tied to the morphological changes seen in depression [3,5]. Leptin directly influences the transport of NMDAR to the neuronal membrane and the formation of new glutamatergic synapses. Thus, leptin modulates synaptic plasticity in the hippocampus and PFC [211,216]. At the same time, by enhancing Ca^2+^ influx in synapses with a high density of NMDARs, leptin increases synaptic formation efficacy [216]. However, in leptin resistance, hyperleptinaemia promotes the heightened density of NMDARs, but the formation of synapses and the functioning of the glutamate system is impaired. This can be detrimental in depressed individuals, since chronic stress, an important player in depression’s neurobiology, can lead to increased glutamate levels [215]. This contributes to glutamate excitotoxity and neuronal atrophy [215]. As a result, this relationship between NMDARs and leptin makes this adipokine a powerful regulator of mood and cognitive processes [211].

The various distribution of LEP-Rs is especially important in depression’s pathophysiology, considering that disrupted leptin signalling in the VTA and NAc, part of the brain’s reward pathway, has been associated with anhedonia, a core symptom of depression [26,217]. Studies on animal models further sustain the association between this adipokine and reward pathway dysregulation, demonstrating that leptin administration drastically reduces social anhedonia [218]. In the VTA, leptin inhibits synaptic transmission, reducing reward-seeking behaviour [219]. At the same time, leptin interacts with the cholinergic neurotransmission to stimulate striatal dopamine release [220]. These actions directly link leptin to social reward and make it a regulator of social behaviour and motivation, behaviours that are impaired in leptin deficiency or leptin resistance [218,219,220].

Obesity is frequently defined by leptin resistance, expressed as high leptin levels that fail to exert their physiological actions [209]. Several mechanisms facilitate this state in the CNS, namely: defects in decreased transport, receptor function, or impaired signalling pathways [209]. Inflammation in the hypothalamus, as well as the activation of resident immune cells, could induce negative regulators of leptin signalling. These are represented in particular by tyrosine phosphatases, such as protein tyrosine phosphatase 1B and T-cell protein tyrosine phosphatase, interfering with normal pathways [210,221]. In the context of impaired leptin transport, it is worth noting that leptin can access the CNS through ways that do not involve the BBB, including tanycytes, pericytes, and choroid plexus routing. Any impairment in their function may be responsible for deficient leptin transport [222,223,224]. Although BBB transport of leptin can be decreased in leptin resistance, recent imaging studies have shown normal BBB function [209,224]. Regarding receptor malfunction, these can be due to either intrinsic abnormalities, such as loss-of-function mutations and trafficking and processing issues, or deficient expression, as induced by a high-fat diet [222,223]. Also, leptin resistance is region-specific rather than global, involving parts of the brain (e.g., the hypothalamus or the hippocampus) afflicted by selective resistance, while others remain unaffected [216].

As far as mood is concerned, this state contributes to emotional dysregulation and anhedonia by decreasing inter-neuronal signalling and synaptic plasticity [23,26]. In such state, neurons expressing LEP-Rs in the *nucleus tractus solitarius* (NTS) present reduced responsiveness to vagal stimulation. As the CNS becomes less sensitive to satiety signals from the digestive tract, food intake increases, and mood disruption is enhanced [25] (Figure 2). Simultaneously, leptin resistance creates a state of hyperexcitability of the hippocampal GABA-ergic system. This disrupts glutamate synaptic formation and promotes glutamate excitotoxicity, known to frequently appear in depression [225].

Leptin inhibits the HPA axis at several points: by suppressing corticotropin-releasing factor from the hypothalamus (thus reducing ACTH secretion by the pituitary gland) and by directly reducing cortisol secretion from the adrenal glands [226]. Diminished inhibitory action of leptin on the HPA axis generates elevated cortisol levels and, thus, an exaggerated stress-response. Subsequently, the dysregulated HPA severely impairs neuronal resilience and plasticity [227]. All of these mechanisms unfold in an already pro-inflammatory background (with elevated pro-inflammatory cytokines, such as IL-1β, IL-6, and TNF-α) that further disrupt neuroplasticity and mood [11,217].

Ultimately, leptin acts as a neurometabolic signal that links adiposity to BDNF-dependent and NMDAR-dependent synaptic plasticity, in addition to reward processing, mechanisms that are disrupted in depression [211,216,217,228]. Intact leptin signalling produces, in fact, antidepressant effects by enhancing hippocampal synaptic formation efficacy [216,228].

Clinically, leptin is strongly associated with atypical depression rather than melancholic depression [229]. This further supports the leptin resistance state that exists in depression [229]. Thereby, leptin fails to control feeding behaviour and mood, leading to key characteristics of atypical depression, such as hyperphagia [229]. Furthermore, circulating leptin is more increased in females compared to males [230]. This suggests that women are more prone to display immunometabolic features, which should be considered when evaluating the treatment strategy [230]. In fact, premenopausal women with depression exhibit higher nocturnal leptin in comparison to their healthy counterparts [231].

Considering this accumulated information, leptin resistance, a condition in which hyperleptinaemia fails to exert the full physiological effects, interferes with mood and cognition, connecting the inflammatory environment specific to depression and anxiety to metabolic disruption. Leptin represents a potential therapeutic target in the treatment of depression, by stimulating hippocampal neurogenesis, decreasing inflammation, and modulating synapsis plasticity [24,26,217]. However, more research is needed to decipher the exact mechanism through which leptin intervenes in depression’s pathophysiology.

### 4.2. Adiponectin

Adiponectin, a potent anti-inflammatory adipokine, is mainly released by white adipocytes. It presents a secretion pattern that is opposite to leptin’s, meaning that its levels decrease as adipose tissue increases [141,232]. Once secreted, adiponectin, in its small isoform, passes the BBB and exerts its functions via two types of receptors with different functions and expression patterns: AdipoR1 and AdipoR2 [233,234]. Several key areas for mood and cognition express adiponectin receptors, such as the VTA, dorsal raphe nucleus, PFC, hippocampus, and amygdala [141]. Through its receptors, adiponectin is involved in depression’s neurobiology by various mechanisms: intracellular signalling pathways, neurotransmitter modulation, synaptic modelling, anti-inflammatory and anti-oxidative actions, and neural metabolism regulation [235,236,237,238,239].

**Figure 3:**
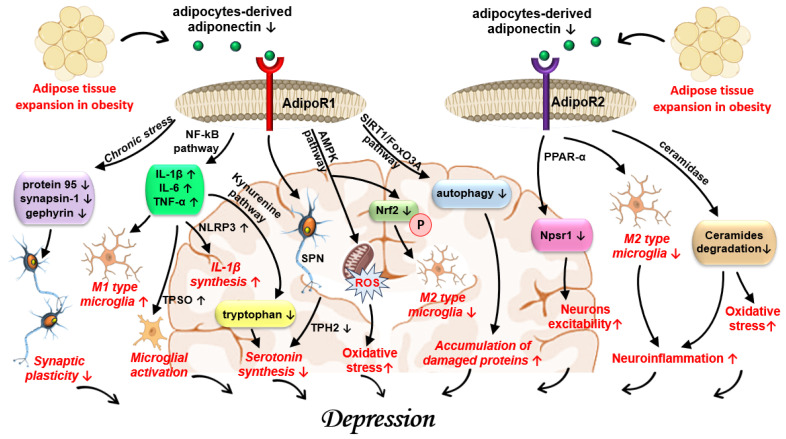
Potential mechanisms of adiponectin in the neurobiology of depression in obesity. AdipoR—adiponectin receptor; IL—interleukin; NLRP3—nucleotide-binding domain, leucine-rich–containing family, pyrin domain–containing-3; Npsr1—neuropeptide S receptor 1; Nrf2—nuclear factor erythroid 2-related factor 2; PPAR-α—peroxisome proliferator-activated receptor α; ROS—reactive oxygen species; SPN—serotonin-producing neurons; TPH2—tryptophan hydroxylase 2; TPSO—translocator protein; ↑—increase; ↓—decrease; →—activation.

AdipoR1 is densely expressed in the hippocampal dentate gyrus (DG) and in the dorsal raphe nucleus [235,239]. In the DG, AdipoR1 is expressed by the neural progenitor cells, where it activates the 5’AMP-activated kinase (AMPK) pathway, facilitating cell proliferation and neurogenesis [239] (Figure 3). Another intracellular cascade activated by adiponectin is that of p38 mitogen-activated protein kinase (p38-MAPK) [234,240]. This pathway supports the functional maturation of new neurons and improves hippocampal synaptic connectivity by positively influencing dendritic arborisation and spinogenesis [234,240]. In the dorsal raphe nucleus, AdipoR1 is highly expressed in serotonin-producing neurons. Within these neurons, it is co-localised with TPH2, an enzyme that is paramount for serotonin synthesis [235]. Disruption of adiponectin signalling in these neurons reduces the synthesis of serotonin and alters the expression of the serotonin transporter [235,241]. Furthermore, adiponectin might counteract the effects of the tryptophan–kynurenine metabolic pathway, which, if stimulated by inflammatory cytokines, reduces the availability of tryptophan for serotonin synthesis. This pathway is significant, as reduced serotonin is considered one of the main mechanisms of depression [236].

Adiponectin’s role in synaptic plasticity and stability has been reported by studies using adiponectin-deficient mice [141,237]. Chronic stress, often observed in depression cases, reduces the expression of AdipoR1 in the hippocampus [237]. This reduction has been associated with diminished expression of synaptic proteins, like postsynaptic density protein 95, synapsin-1, microtubule-associated protein 2, and gephyrin [141,237]. As a result, the formation of dendritic spines decreases, alongside with fewer new functional excitatory and inhibitory synapses in the hippocampus. This creates an imbalance in the excitatory/inhibitory equilibrium and weakens long-term potentiation. This molecular impairment decreases synaptic connectivity and neural communication, producing the ideal background for depressive behaviour [237].

While AdipoR1 is widely expressed in the brain, AdipoR2’s expression is restricted to the dentate gyrus and the hypothalamus [234]. Its activation has been tied to neural responsiveness regulation and contextual fear reduction, processes that are paramount in the pathophysiology of mood disorders [234,236,242]. Adiponectin deficiency renders DG granule neurons hyperexcitable, a mechanism which has been illustrated as slower contextual fear dampening in adiponectin-deficient mice [242]. When these neurons become hyperexcitable, fear responses tend to become more persistent and generalised [243,244]. At the same time, AdipoR2 is coupled with PPAR-α, which modulates neuronal excitability. In this context, AdipoR2’s malfunction might interfere with PPAR-α activation, disrupting its capacity to diminish excitability, thus prolonging the contextual fear [245] (Figure 3). Intersecting with PPAR-α signalling, it is the ceramidase activity of AdipoR2 that allows it to lower membrane ceramides, thereby modulating membrane properties and the functioning of ion-channels [246]. In this regard, stimulation of AdipoR2 by its agonists, such as AdipoRon, exerts a modulatory effect on neuronal excitability and is thought to promote adaptative synaptic remodelling [236].

The anti-inflammatory potential of adiponectin derives from its inhibitory action towards the NF-kB pathway [236,237]. Adiponectin reduces the levels of pro-inflammatory cytokines (IL-1β, IL-6, and TNF-α), diminishing microglial activation and inflammation in the hippocampus and the cingulate cortex, the adiponectin level being inversely correlated with translocator protein (TPSO) binding in these regions [236,247,248]. Also, adiponectin inhibits NLRP3 activation, further decreasing IL-1β synthesis [249]. Along the same lines, adiponectin mediates the polarisation of microglia towards the M2 anti-inflammatory phenotype and lessens the expression of M1 pro-inflammatory microglia [141,248,250]. The adiponectin-mediated activation of the AMPK pathway supports Nrf2 activity, additionally supporting M2 phenotypic shift by increasing antioxidant balance [251]. Moreover, adiponectin can induce autophagy in macrophages and neural cells via SIRT1/FoxO3A axis, preventing the accumulation of damaged proteins and further mitigating depressive neuroinflammation [252].

Adiponectin’s metabolic function must not be disregarded, since neuronal metabolism plays an important part in depression’s neurobiology [11]. By activating the AMPK pathway, adiponectin alleviates neuronal insulin sensitivity, thus supporting the metabolic demands of neurons and the functioning of high energy processes, like neurotransmission and synaptic plasticity. Similarly, adiponectin maintains synaptic integrity by enhancing glucose uptake and mitochondrial biogenesis [253,254]. Additionally, this pathway regulates mitochondrial function and reduces the production of oxygen reactive species, therefore protecting the neurons against oxidative damage from depression [253,254].

The link between adiponectin and metabolic dysfunction is translated in depression clinical features as well [229]. Thus, adiponectin levels are lower in individuals with atypical, metabolically abnormal depression [229]. Contrastingly, no significant differences in adiponectin levels have been reported in melancholic depression [255]. Furthermore, depressed elderly patients present significantly reduced adiponectin compared to controls [256]. This is consistent with a state of adiponectin deficiency that could contribute to the metabolic and cognitive effects of geriatric depression [256].

In summary, a decline in adiponectin levels deprives the CNS of its protective anti-inflammatory effects, facilitating the pathogenesis of depression through several partially deciphered mechanisms [235,236,237,238,239]. Not only does it sustain neuroplasticity and synaptic efficacy, but it also promotes serotonin production and preserves excitatory/inhibitory balance [234,235,236,239,240,245]. Low adiponectin, as seen in obesity, removes its aid for M2 microglial polarisation as well, creating an environment that is detrimental to optimal neuronal function [141,248,250]. Furthermore, adiponectin supports normal neuronal metabolism, contributing to the proper functioning of neurotransmission and synaptic processes [253,254].

### 4.3. Apelin

Apelin is an anti-inflammatory adipokine that exerts its actions through its G-protein-coupled apelin-receptors (APJs) [257,258]. Its corresponding gene encodes for a prepropeptide that can be processed into various isoforms depending on the number of amino acids it contains [27,259]. APJs are heavily expressed in peripheral tissues (adipose tissue, heart, gastrointestinal tract, kidneys) but are also present in the CNS. Within the CNS, APJs are expressed in regions that are central to mood regulation, such as the hippocampus, the hypothalamus, and the amygdala [257,258]. Similarly to other adipokines, apelin intervenes in depressive neurobiology through intracellular signalling pathways, BDNF modulation, NMDAR function regulation, anti-inflammatory potential, and neuronal metabolism [28,257,260,261].

Apelin secretion is insulin-dependent, therefore its levels tend to soar in obesity and hyperinsulinemia [11,262]. In the former, apelin acts as a compensatory hormone that attenuates the co-existing metabolic issues, such as hyperglycaemia, insulin resistance, and hyperlipidaemia [27,262]. Although depression and obesity share a myriad of biological mechanisms, studies exploring apelin levels in depression have been inconclusive so far. Current research has demonstrated that dysregulation of the apelin system is present in depressed patients, with inconsistent apelin levels across different studied populations [259,262,263]. However, the metabolic effects of apelin (increasing insulin sensitivity, glucose uptake, thermogenesis, and normalising lipid profile) are without a doubt beneficial in depression’s pathophysiology [259,262].

In CNS, apelin acts upon three main signalling cascades, namely PI3K/Akt, ERK, and BDNF-TrkB pathways [28,257,264]. PI3K/Akt pathway stimulates the mammalian target of rapamycin (mTOR) to promote synaptic remodelling and resilience, especially in the hippocampus [28]. The activation of the ERK cascade is involved in the neuroprotective actions of apelin, such as vascular endothelial growth factor induction, which provides neurovascular benefits [265]. ERK and BDNF-TrkB pathway activation interferes with three mechanisms that are central to depression’s pathophysiology: neuroinflammation, BDNF downregulation, and HPA axis overactivation [28,264]. Firstly, by stimulating the transcription of BDNF at a nuclear level, it enhances its synthesis, which supports synaptic plasticity and supresses neuroinflammation [28]. Secondly, this pathway modulates GRs’ hippocampal sensitivity, providing apelin with the capacity to normalise HPA axis activity [264].

Apelin also modulates NMDAR activity via several mechanisms [257]. Once apelin interacts with APJs, the activated receptor triggers a molecular cascade that promotes the phosphorylation of the GluN2B component of NMDARs [266]. This causes the receptor’s inability to anchor itself in the neuronal membrane, leading to its endocytosis. The end result is a reduction in NMDAR surface density on the neuronal membrane [266]. Moreover, apelin has been found to reduce NMDA-mediated calcium influx, which prevents intracellular calcium overload, improving synaptic viability in the hippocampus [267]. In addition, apelin inhibits microglial NK-kB/STAT3 signalling, diminishing the production of TNF-α and IL-1β [28,268]. Thereby, apelin averts glutamate diffusion, ultimately decreasing glutamate excitotoxicity, inducing an anti-depressive effect. Furthermore, apelin has potent anti-inflammatory and antioxidant properties. As APJs are expressed on microglia, the effects of their stimulation converge to produce a phenotypic shift in these cells [261]. On the one hand, apelin inhibits the NF-kB/STAT3 pathway, reducing the expression of the M1-pro-inflammatory gene [28,261,268]. On the other hand, apelin upregulates STAT6/PPARG signalling, driving M2 microglia induction [28,261]. Moreover, by acting on these pathways, apelin reduces inducible nitric oxide synthetase (iNOS) expression [261]. Thus, not only does apelin reduce neuroinflammation in depression, but it also protects the CNS from oxidative stress [28,261,268].

All things considered, apelin is a multimodal adipokine. Its secretion and activity are intrinsically linked to the metabolic defects that are common in obesity, such as hyperlipidaemia, hyperglycaemia, and insulin resistance [27,261]. Its direct influence on BDNF synthesis and NMDAR function provide it with neuroprotective abilities, allowing apelin to be a potent mood regulator [28,257]. The neuroprotective potential of apelin is further enhanced by its anti-inflammatory and antioxidant activity, essentially counteracting some of the common mechanisms of depression [28,261].

### 4.4. Resistin

Resistin is an adipocytokine that is generally increased in individuals with depression, especially in those exhibiting an atypical profile of symptoms [10,269]. Its elevation is directly associated with adipose tissue expansion, BMI, and waist circumference. This results in the exacerbation of the inflammatory environment that drives both depression and obesity [10]. High levels of resistin exert detrimental effects on mood via two mechanistic axes: pro-inflammatory and metabolic [270,271,272,273]. In this context, resistin promotes chronic systemic inflammation via several processes. Unlike adiponectin, resistin binds to TLR4 and possibly other pattern-recognition receptors to activate the NF-κB pathway, which induces the production and secretion of TNF-α, IL-6, and IL-12 by macrophages [270,272,273,274]. Consequently, CNS resident immune cells become activated, giving rise to disruptions of mood-related neuronal circuitry [270]. The subsequent neuroinflammation also reduces the expression of BDNF [11]. Further contributing to the pro-inflammatory environment, resistin promotes endothelial cell activation and atherosclerosis, resulting in vascular inflammation. This supplementary inflammation could compromise BBB integrity, facilitating the entry of peripheral cytokines into the CNS, thereby creating a vicious cycle of inflammation [11,270,272].

Once these cytokines reach the neurons, they suppress dopamine and norepinephrine synthesis [269]. Considering that monoamine deficiency is one of the main mechanisms of depression, the predominantly deficient monoamine dictates the symptom profile of the patient [3,5]. Resistin can also be produced by peripheral immune cells, especially neutrophils. The activated neutrophils secrete resistin as a way of communicating with the microglia [241]. The exposure to neutrophil-derived resistin drives the polarisation of the microglia towards the M1 pro-inflammatory type [269,275]. This results in supplementary IL-6 production that further accentuates inflammation [11,270,275].

All things considered, resistin-induced systemic inflammation over-activates the HPA axis, leading to hypercortisolaemia and a dysregulated stress response [269,272]. Resistin levels are positively correlated with free cortisol, illustrating the parallelism between the HPA axis and the resistin system [10]. Conversely, administration of dexamethasone, or hypercortisolaemia, can raise resistin’s expression and levels [276]. Once the HPA axis is over-activated, this dysmetabolic state enters a self-preservation scenario in which low-grade inflammation initiates microglial activation, negatively impacting neuronal function. In sequence, this modifies the sensitivity of brain GRs, basically disrupting the negative feedback of the HPA axis [19].

On a metabolic level, resistin contributes to insulin resistance both in the CNS and peripherally, creating a metabolic environment that predisposes the individuals to depressed mood. This is possible through an alteration of brain energy metabolism, neurotransmitter synthesis, and neuroplasticity [241,271]. In patients with depression comorbid with metabolic complications (e.g., obesity or type two diabetes mellitus), high resistin levels worsen glycaemic control and inflammation [274]. Ultimately, this adds up to resistin’s direct inhibition of dopamine and norepinephrine release, therefore damaging neurotransmission in brain areas involved in affective regulation [277].

In summary, resistin distinguishes itself as a plausible link between the metabolic complications of obesity and the pathogenesis of depression. This adipokine stands at the intersection between metabolism and inflammation, unifying the pathogenic pathways of the disorder. However, results of the studies exploring resistin’s role in the pathogenesis of depression have been inconsistent [10,269]. Considering that resistin might be the key adipokine involved in the development of depression in obese patients [10], further research is needed to clarify the relationship between resistin and the development of depression in metabolically healthy patients and in patients in which inflammatory manifestations are not central.

### 4.5. Chemerin

Chemerin is a pro-inflammatory and proatherogenic adipokine [278]. To become biologically active, chemerin requires proteolytic processing [279]. Its receptors, chemokine like receptor 1 (CMKLR1) and G protein-coupled receptor 1 (GPR1), are expressed throughout the CNS, on adipocyte, and in various immune cells (e.g., macrophages or dendritic cells) [279,280]. Within the CNS, chemerin receptors can be found in areas of importance for mood regulation, such as the hypothalamus, PFC, and the hippocampus [279]. Chemerin’s involvement in the neurobiology of depressive disorder entails pro-inflammatory and neuroendocrine mechanisms [281,282,283,284].

Circulating chemerin levels strongly correlate with BMI and the mass of visceral adipose tissue [285,286]. Physiologically, chemerin promotes adipogenesis, adipocyte differentiation, and angiogenesis in the adipose tissue [286]. Increased chemerin stimulates adipose inflammatory status through supporting macrophage chemotaxis to adipose tissue [281,286]. Additionally, in obese patients, chemerin levels not only predict cardiometabolic complications, but also negatively affect vascular tone and increase oxidative stress [278,286].

Peripherally, chemerin stimulates the activity of immune cells and their ability to produce pro-inflammatory cytokines, such as IL-1β, IL-6, and TNF-α [281]. The adverse inflammatory events that trigger the elevation of chemerin levels also lead to an initial upregulation of CMKLR1 expression [281,284]. However, if such events persist, chemerin expression is downregulated and a wave of neuroimmune activation ensues [284]. The activated CMKLR1 produces the mobilisation of calcium from intracellular deposits and elicits the MAPK pathway, thereby regulating microglial activity to exert neuroinflammatory effects [284,287]. This creates neuronal damage via microglial release of oxygen reactive species and additional cytokines [282].

Paradoxically, chemerin has been found to have CNS anti-apoptotic, anti-inflammatory, and antidepressant effects in various animal models [280,283,288]. Stimulation of CMKLR1 engages the PI3K/Akt pathway and Nrf2 to inhibit the activation of the inflammasome and cell death [288]. At the same time, in a study using recombinant chemerin, the adipokine significantly supressed neuronal apoptosis by stimulating the CaMKK2/AMPK pathway [280]. Moreover, studies using lipid agonists of CMKLR1 have postulated that this receptor engages the mTOR pathway to trigger BDNF release in the medial PFC [283,289]. Loss of neurotrophic support by decreasing BDNF is a major mechanism of depressive disorder and has been associated with morphological changes in the hippocampus and the forebrain [54]. In this context, chemerin’s ability to restore BDNF secretion might be essential for its antidepressant action [283,289].

A recent case–control study showed that chemerin levels of depressed patients were lower than those of healthy controls [290]. Considering the combined depression–obesity phenotype, a clear divergence arises with obesity, sustaining high chemerin levels, while those levels are normally decreased in depression [285,290]. In this instance, chemerin signalling might be mismatched [286]. Peripheral chemerin may sustain inflammation and HPA axis dysfunction, whereas the lower central chemerin may deprive the brain of its neurotrophic support [283,284,286,289].

Not least, the other chemerin receptor, GPR1, co-localises with numerous neuroendocrine regulators. Amongst these, the most significant are CRH and the gonadotropin-releasing hormone (GRH). Hence, chemerin might modulate the levels of certain hormones essential for mood and emotional regulation (e.g., cortisol, oestrogen, CRH, and GRH) [283].

To conclude, chemerin’s actions differ centrally and peripherally, yet cause increases in pro-inflammatory cytokines that can induce neuroinflammation and activate immune cells [264,284]. However, recent studies illustrate the neuroprotective potential of chemerin, which could be the basis for future antidepressive treatments [280,283,289]. In this respect, translational research is needed to ascertain chemerin’s therapeutic prospects for depressive disorder.

### 4.6. Omentin

Omentin is another anti-inflammatory adipokine, primarily secreted by the stromal-vascular compartment of visceral adipose tissue [291,292]. Omentin levels are decreased in both depression and obesity, suggesting that this adipokine might control shared biological mechanisms [291]. Omentin has four main mechanisms of action in terms of depression’s pathophysiology: anti-inflammatory, barrier enhancement, metabolic, and anti-oxidative [11,291,293].

The anti-inflammatory activity of omentin resides in its ability to downregulate the production of IL-1β, IL-6, and TNF-α, which are often involved in depression’s pathogenesis [10,291]. This action is performed either directly or by engaging integrin receptors αvβ-3 and αvβ-5 on macrophages [291]. Thus, omentin intervenes by normalising neurotransmitters’ balance and improving neuroplasticity, thereby alleviating depressed mood and cognitive deficits [291,293].

A possible contributor to the anti-inflammatory effects of omentin might be its ability to support endothelial barrier integrity [291]. Omentin acts upon the PI3K/Akt pathway, which stimulates revascularisation and the tightening of intercellular junctions [294,295]. Considering that BBB breakdown is directly correlated with neuroinflammation, omentin’s role in maintaining barrier integrity might have protective effects against depressive inflammation [294,295,296]. However, there is insufficient data to support omentin’s protective role on the BBB.

Omentin upholds the neurochemical homeostasis of the brain by modulating neuronal metabolism. Omentin increases insulin-mediated glucose uptake and insulin sensitivity, promoting energy production in neurons [11]. Therefore, omentin indirectly provides the neurons with sufficient energy to sustain normal neurotransmission [297]. It also activates a central metabolic pathway, the Akt/mTOR pathway, leading to improved neurogenesis [11]. Thus, omentin exerts its antidepressant effect by restoring neurotrophic support [297]. This promotes the differentiation of neural stem cells and their integration into existing neural circuits [11].

Omentin’s anti-oxidative action is linked to the activation of the Nrf2 pathway [291]. Peripherally, omentin activates Nrf2 with the inhibition of NF-kB pathway and the expression of antioxidant enzymes, such as superoxide dismutase (SOD) [298]. This, combined with the activation of the Akt pathway, offers omentin synaptic–protective proprieties, enabling synaptic transmission and plasticity [11,291].

In conclusion, omentin’s anti-inflammatory, antioxidant, and prometabolic properties illustrate its antidepressant potential, targeting some of the most common mechanisms of depression’s pathophysiology [11,291,298]. In fact, omentin might even prevent the advent of pro-inflammatory cytokines by enhancing BBB integrity—this hypothesis opens an intriguing area of research [291]. All in all, new studies are necessary to ascertain the relationship between omentin and depression in obese patients and to elucidate its exact neurobiological mechanisms.

### 4.7. Visfatin

Visfatin (nicotinamide phosphoribosyl transferase, NAMPT) is an adipokine released by visceral adipose tissue, muscle, and immune cells [274]. Compared to other adipokines, visfatin can be found in two forms: extracellular visfatin (eNAMPT) and intracellular visfatin (iNAMPT). Its extracellular form acts as a pro-inflammatory cytokine that promotes the secretion of IL-6, TNF-α, and IL-1β, molecules that are closely linked to depressive disorder [10,299]. Its intracellular counterpart is a rate-limiting enzyme in NAD synthesis [299].

A significant elevation of eNAMPT serum levels is registered in depressive disorder, especially in elderly people [300,301]. Given that depression is characterised by a state of chronic low-grade inflammation, it has been suggested that eNAMPT contributes to this state, by increasing inflammatory cytokine production [299,301]. At the same time, chronic stress stimulates cortisol secretion, which, in turn, upregulates NAMPT expression [299]. Therefore, an overactive HPA axis, a common mechanism of depression, might aggravate depressive disorder by raising eNAMPT levels [11,299,301]. At the same time, eNAMPT induces iNOS, boosting oxidative stress [299,301]. This is especially important, since further research supports the contribution of oxidative stress and mitochondrial dysfunction to depression’s pathophysiology [10,11].

The iNAMPT is directly involved in energy production by modulating a rate-limiting step in NAD production. Thereby, iNAMPT activates sirtuin-1 (SIRT1), which suppresses NF-kB to limit inflammation and pro-inflammatory cytokine production (TNF-α, IL-6, and IL-1β) [299]. Loss of iNAMPT via genetic manipulation led to depressive behaviour and cognitive decline in murine models. Consequently, it lowered the transcription factors necessary for neurotrophic genes, such as BDNF and reduced monoamine neurotransmitters (e.g., serotonin, dopamine, and norepinephrine) [302]. These anomalies were reversed once NAD levels returned to normal, illustrating the role of iNAMPT in mood regulation and cognition [302].

Metabolically speaking, NAMPT can bind to a site on the insulin receptor that allows it to mimic insulin’s activity. The visfatin–insulin receptor bond triggers multiple intracellular cascades to promote glucose uptake and its storage in the liver and muscle [299,303]. However, elevated visfatin levels, as seen in obesity, have detrimental effects on metabolic health, predisposing the patient to cardiometabolic complications, such as atherosclerosis and arterial hypertension [301,303]. Additionally, visfatin is able to cross the BBB and reach the hypothalamus [304]. In this mood-regulating region, high levels of visfatin can induce cytokine production, leading to sickness behaviour that might precede or overlap with depressive symptomatology [300,304]. Simultaneously, heightened levels of visfatin have a proclivity to promote ROS production and oxidative stress, contributing to the dampening of hippocampal neurogenesis from depression (Table 3) [299,302]. At the same time, higher visfatin levels are a sign of improper glucose utilisation, both peripherally and in the CNS. This phenomenon is common in depressed patients that exhibit poor glucose uptake in the frontal cortex [305]. Visfatin’s metabolic activity might influence neuronal metabolism and exacerbate energy deficits, worsening mitochondrial dysfunction in depression [302,305]. As a result, considering the combined depression–obesity phenotype, visfatin acts not only as a metabolic regulator, but also as a marker of metabolic stress [299,301,303].

Taken together, visfatin intervenes in the pathophysiology of depression, either through its pro-inflammatory effects or through the modulation of energy production. Its pro-inflammatory activity decreases neuroplasticity by lowering neurotrophic support [299,301]. Its levels are regulated by the HPA axis, which is overactive in depression, making visfatin a marker not only of mood, but also of stress response [11,299,301]. Finally, visfatin regulates neuronal metabolism in a dose-dependent manner, either promoting energy synthesis, in physiological levels, or increasing oxidative stress [302].

## 5. Depression Treatment in the Relationship with Adipokines

The pharmacological treatment of depression represents one of the three therapeutic axes used in its management (lifestyle changes, psychotherapy/counselling, and pharmacotherapy) [2,30]. Antidepressants (ADs) aim to correct the neurochemical imbalances found in the depressive brain, thus restoring brain homeostasis [306]. While conventional AD medication addresses certain neurotransmitters’ systems afflicted in depressive disorder, there is significant data that pinpoints their effects on adipokines, most notably adiponectin, leptin, and resistin. ADs’ anti-inflammatory effects are drug-specific, with each drug targeting different inflammatory mechanisms involved in depressive pathophysiology [307]. Notwithstanding, long-term immunological shift and microbial actions might counteract the Ads’ anti-inflammatory actions [308]. However, the existing evidence is still sparse, additional research being needed to establish the mechanisms and the effects of ADs on adipokine levels [309].

The mechanisms that are involved in ADs’ modulation of adipokine levels are various, representing a complex molecular interplay. They target main pathogenic components of depression (HPA axis, BDNF production, and inflammation), but changes in adipokines mainly involve modifications in body weight and metabolic state [310,311]. Moreover, several factors determine how ADs impact adipokine levels, including patient characteristics (such as BMI and baseline metabolic state), duration of treatment, dosage, and, most importantly, the specific AD class [312].

Thus, selective reuptake inhibitors (SSRIs) (sertraline, fluoxetine, paroxetine, escitalopram, and citalopram) block the serotonin transporter (SERT), allowing for an increase in serotonin in the synaptic cleft, while also interacting with BDNF signalling [313]. Fluoxetine has been demonstrated to increase BDNF expression and improve leptin sensitivity in the hypothalamus [310]. Interestingly, short-term treatment with SSRIs does not modify circulating leptin levels, suggesting that their actions do not need hyperleptinaemia to enhance leptin signalling [310,314]. As far as adiponectin is concerned, SSRIs reduce neuroinflammation, resulting in the improved function of AMPK/PPARG cascades, which are key mechanisms of adiponectin’s action [315]. Yet it should be noted that chronic AD treatment has been reported to cause some degree of circulating adiponectin decrease. Resistin level also declines on SSRI treatment, concomitantly with HPA axis normalisation and decreased neuroinflammation [309]. Moreover, SSRI therapy leads to a reduction in IL-6, IL-8, IL-10, and MCP-1, in direct correlation with symptomatic remission [316]. Mechanistically, SSRIs produce a shift in cytokine balance toward an anti-inflammatory profile via NF-κB inhibition [317].

Serotonin and norepinephrine reuptake inhibitors (SNRIs), such as venlafaxine and duloxetine, raise synaptic serotonin and norepinephrine. Simultaneously, they support peripheral beta-adrenergic signalling that promotes lipolysis and consequent moderate leptin reduction [313]. Like SSRIs, no significant increase in circulating leptin has been observed on short-term SNRI treatment [314]. Furthermore, SNRIs generally exhibit anti-inflammatory proprieties, their administration being linked to reductions in IL-6 and TNF-α [307]. Venlafaxine, in particular, acts upon brain chemokine networks (CXCL12/CXCR4/CXCR7 and CX3CL1/CX3CR1), tuning them to their physiological functions [318].

Tricyclic antidepressants (TCAs) (e.g., amitriptyline, clomipramine, imipramine, and nortriptyline) disrupt SERT and norepinephrine transporter functioning, while blocking various receptors, such as histamine receptor-1 (H1), muscarinic receptor-1 (M1), and alpha-adrenergic receptor-1 [314]. Their potent anti-H1 activity promotes hyperphagia and weight gain, which, in turn, leads to hyperleptinaemia with central leptin resistance. For instance, a 4- to 5-week course of treatment with amitriptyline resulted in a 40% to 60% increase in leptin [314]. Additionally, their dysmetabolic effects result in adiponectin suppression due to increased appetite [319]. Regarding inflammatory pathways, there is an important drug-specific heterogeneity within the class [307]. Generally, TCAs have fewer anti-inflammatory effects than SSRIs or SNRIs, yet some examples, like clomipramine, seem to decrease IL-6, IFN-γ, and TNF-α [317].

Mirtazapine is an atypical AD that targets several neurotransmitters, including serotonin, histamine, and norepinephrine [314]. In this respect, mirtazapine has been demonstrated to inhibit α2-adrenergic and H1 histamine receptors located on the tuberomammillary nucleus of the hypothalamus, which may contribute to increased appetite by upregulating neuropeptide Y (NPY) and ghrelin activity [320]. Moreover, its 5-HT2 antagonism further contributes to weight gain [320]. This is further reflected in mirtazapine’s effects on the production increase of pro-inflammatory cytokines (TNF-α and IL-6) [317]. For these reasons, patients with an immunometabolic pattern of depression should be carefully monitored, and an alternative therapy should be considered [314].

Another atypical AD, bupropion, has opposite effects to mirtazapine, promoting either weight loss or weight neutrality [321]. Its mechanism involves the inhibition of dopamine and norepinephrine reuptake, which allows bupropion to support a balanced dopamine/adrenergic tone [321,322]. Consequently, bupropion suppresses NF-kB activity, which leads to lower resistin and anti-inflammatory actions that enhance AD response in immunometabolic cases [322]. Bupropion administration has been linked to a Th1 to Th2 shift, by decreasing the pro-inflammatory cytokines (e.g., IFN-γ, TNF-α, and IL-1β), while supporting IL-10 secretion [323]. Its weight profile signifies lowered leptin and heightened adiponectin by reducing adipose tissue’s mass and improving insulin sensitivity [321].

Some newer ADs also have potential metabolic features. Vortioxetine, a multimodal AD, acts on 5-HT1A and 5HT-7 receptors in the brain, enhancing cognition and self-care behaviour that can indirectly improve adipokine profiles [324]. At the same time, it presents significant influence on body weight, which normalises leptin and adiponectin levels [325]. Consequently, vortioxetine proved to be more effective than sertraline in type 2 diabetic patients in improving metabolic control, which was associated with lower systemic inflammation [325]. Additionally, vortioxetine reduces IL-6 and TNF-α, highlighting its anti-inflammatory effects, similarly to other serotonergic compounds [308]. The melatonergic agonist agomelatine has been shown to improve insulin resistance and reduce obesity associated with a high-fat diet in rodent models [326]. Furthermore, agomelatine has anti-inflammatory (reductions in TNF-α, IL-1β, and IL-6) and pro-metabolic characteristics that create a favourable adipokine profile, with reduced leptin resistance and increased adiponectin [326,327]. Interestingly, agomelatine appears to have renoprotective effects in metabolically unhealthy obese animal models via its cytokine-reduction proprieties [328].

Most notably, studies on ketamine, a rapid-acting AD for treatment-resistant depression, have shown that it can reduce resistin levels, increase adiponectin levels, and attenuate the severity of depressive symptoms [272]. In addition to the rapid symptom improvement, ketamine induces fast IL-6 and IL-1α reduction [329]. This might be due to ketamine’s rapid action, which increases BDNF and mTOR pathways [308].

Promising treatments for depressive disorder have started to be investigated. Probiotics may induce small to moderate reductions in CRP and TNF-α, while increasing BDNF, with neurotrophic improvements in depressed patients [330,331]. Faecal microbiota transplantation proved to be safe and feasible for depressed patients in a randomised controlled trial reporting moderate improvement of depressive symptomatology [332]. Certain dietary patterns, i.e., the Mediterranean diet, modulate the microbiome, while simultaneously improving depressive symptoms, as shown in the SMILES trial [333]. Additionally, adipokine-based therapies (e.g., Metreleptin and AdipoRon) might prove effective in the treatment of depression, especially when associated with obesity [26,236]. However, large randomised controlled trials are needed to ensure safety, efficacy, and to identify the contraindications.

Above all, adipokine levels might serve as a prognostic factor for AD treatment efficacy. In this respect, patients with an elevated level of adiponectin had fewer clinical improvements on different AD classes, including ketamine, regardless of variations in other clinical factors (severity, BMI, age, or gender) [334]. Yet results tend to vary when depression overlaps with other psychiatric comorbidities, such as generalised anxiety disorder, where higher adiponectin might predict favourable outcomes [334]. Leptin’s predictive role is difficult to assess, given the frequency of leptin resistance in depressive disorder. For this reason, while some studies report improved therapeutic results for patients with higher leptin levels, data indicates that individuals with other physical ailments show the opposite effect [335]. Unfortunately, the prognostic role of other adipokines (e.g., apelin or visfatin) has not been yet established.

In conclusion, there is currently a wide array of options for the treatment of depression, with each class of medication being prescribed according to the patient’s clinical profile [2,30]. The effects of AD therapy on adipokine levels are class- and even drug-specific, since some drugs, i.e., mirtazapine, tend to favour appetite and allow more weight gain [310,311,314,320]. Although data regarding the relationship between ADs and certain adipokines (leptin, adiponectin, apelin, and resistin) are abundant, there is an evident scarcity of evidence regarding other adipokines, needing significant supplementary research.

## 6. Futures Perspective of Adipokines-Based Therapy in Depression

Considering the existing evidence connecting altered adipokine signalling to depressive neurobiology, several pharmacological interventions that aim to restore or mimic adipokine activity have been devised. Emerging treatments targeting adipokines attempt to modulate mood by correcting upstream metabolic and inflammatory events that contribute to the development and persistence of depressive disorder [26,27,28,29]. Even though most available data come from preclinical models, early clinical research suggests that such therapies may offer a distinct biological adjunct to conventional ADs.

Metreleptin, a recombinant human leptin analogue, was developed for the treatment of leptin deficiency, such as congenital leptin deficiency and lipodystrophy [336,337,338]. Peripherally, it regulates the metabolic profile by ameliorating plasma glucose and serum triglycerides [337]. Centrally, it retains high affinity to LEP-Rs, duplicating leptin’s activity on intracellular pathways (JAK/STAT3, AMPK, and ERK1/2) [337,339,340]. Moreover, Metreleptin appears to have anti-inflammatory properties, counteracting the effects of commonly found depressive neuroinflammation by reducing the levels of pro-inflammatory cytokines in the PFC and the striatum [26]. While there is no direct evidence for its role in depressive disorder, in *anorexia nervosa* patients, the restoration of leptin signalling by metreleptin has been associated with a quick correction of both weight and mood [341]. In lipodystrophy cohorts, Metreleptin’s mood-alleviating properties were linked to an improved quality of life [338,342]. Reports have stated that its administration resulted in a striking amelioration of depression scores and mood [338,343,344]. Yet leptin resistance might hinder metreleptin’s antidepressive effects, so its co-administration with substances counteracting this phenomenon, such as celastrol and withaferin A, might prove to be beneficial [345].

Intracerebroventricular administration or adenoviral delivery of adiponectin were linked to significant reductions in depressive-like behaviours in preclinical models [250,346]. Furthermore, they also led to diminished behavioural despair and reduced microglial activation, which are common findings in depressed subjects [250,346]. Pharmacological manipulation of adiponectin signalling through adiponectin receptor agonists, i.e., AdipoRon, demonstrated swift antidepressant and anxiolytic-like effects, especially in metabolically impaired subjects [236]. Alterations in neuronal excitability and neurotransmission in the amygdala and PFC were identified to be major contributors to these effects [236,347]. Other adiponectin-related molecules, such as ADP355 and ALY688, have been shown to stimulate AdipoR1 and AdipoR2 [348]. This allowed for the activation of adiponectin’s downstream signalling pathways, i.e., PI3K-Akt/AMPK, indicating that these compounds were able to replicate the physiological mechanisms of adiponectin [348]. Additionally, they were correlated with anti-inflammatory shifts, further supporting their potential role in immunometabolic depression [349].

Administration of apelin-13, an isoform of apelin, restored inhibitory feedback of the HPA axis in rodents by normalising elevated corticosterone levels and promoting GR nuclear translocation [264]. The upregulation of BDNF and the activation of its specific receptor are assumed to be partially responsible for these effects, which in turn would impact regulation of the HPA axis [28]. At the same time, apelin-13 administration has been shown to reduce neuronal apoptosis and neuroinflammation and improve cognitive function and mood in these animals [27]. Longer-acting apelin analogues, i.e., LIT01-196, were demonstrated to have similar effects [264]. Recent pharmacological advances permitted the development of other stable leptin analogues, paving the way for potential new methods to influence the brain’s circuitry [253]. However, no large-scale clinical trials have been carried out so far.

The blockade of chemerin’s CMKLR1 by its antagonist, α-NETA, reduces brain inflammatory events, rendering it a potential candidate for the immunometabolic depression phenotype [29,350]. Mechanistically, this molecule inhibits chemerin signalling and reduces chemoattraction, diminishing the influence of the inflammatory milieu over brain cells [350].

The role of inflammation in the pathogeny of this combined phenotype is also confirmed by pharmacological studies that have explored the effect of cytokine blockade on both disorders [351]. Evidence from studies concerning systemic inflammation in various disorders has concluded that anticytokine biological therapy possesses small to modest antidepressant potential [352]. Firstly, lifestyle interventions, such as reduced caloric intake, certain diets, and increased physical activity, have proven to be effective in reducing pro-inflammatory cytokine levels and CRP, while improving metabolic outcomes and mood at the same time [353,354]. Moreover, infliximab, an anti-TNF-α agent, demonstrated clinical psychiatric improvements in individuals with increased systemic inflammation [355]. Biological therapy, i.e., IL-6 receptor antagonists, has shown promising AD effects, alongside weight improvement [351]. In experimental models as well as in rheumatoid arthritis cohorts, Il-6 inhibition by tocilizumab resulted not only in a decreased symptom burden, but also in a reduction in depressive risk [356]. Furthermore, anti-cytokine interventions have been found to perform better than placebo on depressive scores, indicating the need of AD treatment tailoring according to patients’ cytokine profiles [355].

Some antidiabetic medications, such as glucagon-like peptide-1 (GLP-1) receptor agonists and glucose-dependent insulinotropic polypeptide (GIP)/GLP-1 dual agonists, may bridge metabolic and psychiatric health [357,358,359]. GLP-1 receptor agonists and GIP/GLP-1 dual agonists provide a significant reduction in cardiovascular events by improving weight and glucose metabolism [357,358]. Added with a significant weight reduction and cardioprotective effects, these drugs induce a decrease in depressive symptoms [359]. This effect might be attributed to their capacity to enhance hippocampal neurogenesis and to prevent neuroinflammation [359]. Therefore, GLP-1 receptor agonists and GIP/GLP-1 dual agonists succeed in treating several aspects of the patients’ health, representing a first step towards a fully integrated treatment strategy for depression comorbid with metabolic disorders [357,358,359].

Although preclinical data appear compelling, translating adipokine-based therapies into safe and feasible treatment alternatives for depression faces multiple challenges.

Firstly, the BBB limits the CNS access of adipokines or their analogues [360]. Moreover, adipokine transporters can become saturated or altered in obese individuals [360]. Leptin transport is deficient in obesity, as a landmark of leptin resistance, whereas adiponectin penetration through the BBB is highly variable [360,361]. This could lead to pharmacological correction of adipokine levels and their inefficient signalling, since adipokine analogues cannot reach the CNS [360,361]. For this reason, adipokine analogues might require higher doses or alternative administration routes (i.e., intranasal) to achieve CNS-relevant effects [362,363].

Secondly, pharmacokinetics optimisation represents a significant challenge in the development of new therapies. This is especially true for adipokines that have a short half-life (i.e., apelin) [362]. Thus, an apelin-derived molecule with an extended half-life owing to a fluorocarbon addition, LIT01-196, has been developed, suitable for systemic administration [362]. In this context, LIT01-196 has been shown to have a beneficial cardiovascular effect (e.g., improved cardiac function and decreased blood pressure) in an experimental model [362]. However, when repurposed for depression, it might negatively influence fluid balance, organ perfusion, and blood pressure. Moreover, LIT01-196 tissue distribution could interfere with its safety parameters, so further clinical characterisation is still necessary [362].

Lastly, adipokines are multimodal molecules that regulate appetite, glucose metabolism, immune response, and cardiovascular function [248]. In this respect, dosing adipokine-based treatments might perturb metabolic or vascular systems [248]. Metreleptin rapidly improves depressive symptoms in anorexia nervosa, yet it alters weight and energy metabolism [364]. For this reason, clinicians must be aware of potential metabolic side-effects that might exceed Metreleptin’s psychiatric benefits [364].

Finally, adipokine-centred therapy has garnered the interest of researchers as a potential new treatment for patients with depression that present modified adipokine profiles. So far, only Metreleptin has gathered enough evidence to support its mood-improving proprieties in humans, although no direct data in patients with depressive disorder is currently available [338,341,343,344]. Preclinical evidence is available for the pharmacological manipulation of other adipokines, such as adiponectin, apelin, and chemerin, but robust randomised clinical trials are needed to confirm the efficacy of such treatments in humans [29,236,264,347]. With regard to inflammatory adipocytokines, while improvements in mood were noted, it is not yet clear whether they are due to an amelioration of the underlying disease or a direct effect on depressive neurobiology [351,353,354,355].

Despite providing an integrative overview of the molecular links between adipose tissue and depression, this study has several limitations. First and foremost, most of the available data comes from preclinical or cross-sectional studies, limiting our ability to determine the causality between adipokine dysregulation and depressive symptoms. Secondly, considerable heterogeneity exists in clinical studies in terms of diagnostic criteria, sample characteristics, and assessment methods, rendering direct comparison and meta-analytic synthesis complicated. Lastly, publication bias toward positive findings and the limited representation of diverse populations may restrict the generalisation of current evidence. As a result, further longitudinal, multimodal, and omics-based studies are needed to validate the proposed immunometabolic pathways and clarify their therapeutic relevance in depression associated with obesity.

## 7. Conclusions

The bidirectional relationship between depression and obesity has been exhaustively illustrated by epidemiological, genetic, and mechanistic studies. This reciprocity initiated a shift in psychiatric research from a neurotransmitter-centred view of depression towards an integrative understanding that includes immunometabolic and endocrine factors.

Adipokines have been at the forefront of the adipose–brain crosstalk research in recent years as key mediators for this relationship. Among these adipose-secreted factors, leptin and adiponectin are the best-described mood regulators, while other adipokines (resistin, apelin, omentin, and chemerin) have not yet been thoroughly described in terms of their relationship with mood. By interacting with their receptors, adipokines regulate crucial bodily functions and ensure optimal activity of the brain–adipose–gut axis. Dysregulated adipokines signalling maintains a dysmetabolic and pro-inflammatory environment that is detrimental to mood and that sustains the effects of obesity. Therefore, adipokines act as a biochemical bridge between peripheral metabolic imbalance and central mood disorders.

The paradigm shift towards an immunometabolic concept of depression paves the way to new therapeutic opportunities. Preclinical and early clinical research has shown promise for treatments targeting inflammation, adipokine signalling, and metabolic balance. Interventions that restore gut microbiota diversity or dietary interventions may be complementary to the conventional neurochemical approach. Thus, we suggest a multimodal strategy that highlights the potential of adipokine-based and metabolic treatments adjunct to classical antidepressant therapy.

Currently, there are numerous gaps in our understanding of how different adipokines are involved in the pathogenesis of depression. Therefore, more research is needed in order to better grasp how adipokines interact with the BBB, the signalling differences and effects on different demographics, and the mechanisms of their involvement in other pathogenic theories of depression (e.g., HPA axis overactivation, gut dysbiosis, and mitochondrial dysfunction). Additionally, the cumulated research efforts would lead to novel interdisciplinary treatment strategies and to the identification of new compounds that may be beneficial for patients with ineffective conventional antidepressive therapy response.

Our narrative review aimed to offer a better understanding of adipose–brain interactions, which have become a critical boundary in depression research. Integrating inflammation, neuroendocrine, and metabolic dysfunctions might provide a unifying framework that explains the combined depression–obesity phenotype. Understanding how adipokines and other molecules intervene in this dialogue is essential for developing new innovative adipokine-based treatment strategies that target both mood and metabolic health. Approaching depressive disorder as an immunometabolic disorder rather than a neurochemical one might be crucial for creating more effective, personalised, and interdisciplinary interventions for the most significant predicted contributor to global disease burden by 2030.

## Figures and Tables

**Table 1:** The features of inflammatory endotypes identified in depression.

Inflammatory Endotype	Biomarkers	Clinical Features	References
Immunometabolic depression	↑ hsCRP ↑ IL-6, TNF-α ↑ leptin, insulin ↓ adiponectin	Atypical symptoms (hyperphagia, weight gain, and hypersomnia)	[126,128]
Inflammation-related dopaminergic dysfunction	↑ hsCRP ↑ IL-6 ↑ sICAM-1	Anhedonia Reduced motivation Reduced psychomotor activity	[127]
Type-I interferon inflammatory depression	↑ IFN-gene activation ↑ CXCL10 ↑ Kynurenine-to-tryptophan ratio	Dopaminergic effects (anhedonia and psychomotor activity ↓)	[132,133,134]
Early-life-stress inflammatory profile	↑ hsCRP ↑ IL-6 ↑ glucocorticoids	Exposure to ELT Sickness behaviour	[56,135,136]
Glial neuroinflammation	↑ TSPO binding on PET scans ↑ IL-6 in CSF	Higher probability of suicidal ideation	[129,130,131]
Adaptative immunity Th17 profile	↑ IL-17A ↑ Th17-to-Treg ratio	Suicidal ideation Social withdrawal (stress-reactivity ↑)	[7,137,138]
Low-grade inflammatory depression	hsCRP < 1 mg/L	Improved response to selective serotonin reuptake inhibitors	[139]

CSF—cerebrospinal fluid; CRP—C-reactive protein; CXCL10—C-X-C motif chemokine 10; hsCRP—high-sensitivity CRP; IFN—interferons; IL—interleukin; PET—positron emission tomography; sICAM-1—soluble intercellular adhesion molecule-1; Th—T helper lymphocytes; TNF-α—tumour necrosis factor α; TSPO—translocator protein; ↑—increase; ↓—decrease.

**Table 2:** The main studies exploring the brain–adipose tissue–gut axis in depression and obesity.

Study Design	Participants	Main Findings in Depression and Obesity-Associated Disorders	Perspective Linking Obesity to Depressive Disorder	References
Cross-sectional study	11,324 adult patients	Systemic inflammation	NLR ↑ SIRI ↑ SII ↑	[20]
Cohort study	9217 adult patients	Developing DEP symptoms in women compared to men	Managing unfavourable lifestyle factors Implementation of preventative strategies	[48]
Cohort study	889 paediatric patients	Early childhood obesity increases the DEP risk in later life	Additional studies exploring childhood obesity	[84]
Meta-analysis and Mendelian randomisation study	371,184 patients with DEP and 806,834 obese patients	16 genomic loci are significant in both BMI and DEP	Higher genetic risk for depression leads to higher BMI and vice versa Development of novel therapeutic targets	[85]
Meta-analysis	6902 DEP patients and 6799 controls	DEP increases the effect of the FTO gene on BMI	Understanding the linking biological mechanisms	[92]
Multicentre randomised controlled trial	136 DEP patients and 1987 controls	Non-genetic risk factors, i.e., BMI, added to GRS may improve DEP prediction	New therapeutic interventions	[117]
Experimental study	5-week-old male C57 mice	Allicin reduced depressive-like behaviours in obesity	ROS ↓ Mitochondrial damage ↓ Autophagy ↓ Insulin resistance ↓ Potential therapeutic agent	[200]

BMI—Body mass index; DEP—depression; FTO—Fat mass and obesity-associated protein; GRS –genetic-risk score; NLR—Neutrophil-to-lymphocyte ratio; SII—Systemic immune-inflammation index; SIRI—Systemic inflammation response index; ↑—increase; ↓—decrease.

**Table 3:** Potential role of other adipokines in the association between obesity and depression.

Adipokines	Mechanisms	Effects	References
Apelin	PI3K/Akt, ERK, BDNF-TrkB, NK-kB/STAT3, and STAT6/PPARG pathways	NMDAR expression ↓ glutamate diffusion ↓ iNOS expression ↓ TNF-α and IL-1β ↓ microglial cells activation ↓ synaptic remodelling ↑ BDNF synthesis ↑ vasoactive effects ↑	[28,257,264,268]
Resistin	TLR4/NF-κB, p38MAPK, and 5-STATS pathways	BDNF ↓ HPA axis ↑ insulin resistance ↑ TNF-α, IL-6, and IL-12 ↑ BBB integrity ↓ dopamine and norepinephrine synthesis ↓	[11,270,272,273,274]
Chemerin	MAPK pathway	microglial activation ↑ TNF-α, IL-6, and IL-1β ↑ ROS ↑ HPA axis ↑	[281,282,285,289,290]
	PI3K/Akt, CaMKK2/AMPK, and mTOR pathways	BDNF ↑ TNF-α, IL-6, and IL-1β ↓ neuronal apoptosis ↓ CRH secretion ↓ GRH secretion ↑	[280,281,283,288,289]
Omentin	PI3K/Akt, Akt/mTOR, and NF-kB/ Nrf2 pathways	plasticity ↑ BBB integrity ↑ TNF-α, IL-6, and IL-1β ↓ neurons metabolism ↑ synaptic plasticity ↑ neurogenesis ↑	[11,291,294,295,296,298]
Visfatin	PI3K/Akt and NAD+ / SIRT1 pathways	HPA axis ↑ neuroplasticity ↓ neuroprotection ↓ glucose uptake in CNS ↓ TNF-α, IL-6, and IL-1β ↑ ROS ↑	[10,11,299,302,305]

BBB—blood–brain barrier; BDNF—brain-derived neurotrophic factor; CNS—central nervous system; CRH—corticotropin-releasing hormone; GRH—gonadotropin-releasing hormone; HPA axis—hypothalamic–pituitary–adrenal axis; iNOS—inducible nitric oxide synthetase; IL—interleukin; NMDAR—*N*-methyl-d-aspartate receptor; ROS—reactive oxygen species; TNF-α—tumour necrosis factor α; ↑—increase; ↓—decrease.

## Data Availability

No new data were created or analysed in this study.
